# Subsequent epilepsy surgery after SEEG-guided radiofrequency thermocoagulation: Outcomes and prognostic value of treatment response—a systematic review and meta-analysis

**DOI:** 10.1007/s10143-026-04561-x

**Published:** 2026-11-06

**Authors:** Amirmahdi Mojtahedzadeh, Seyedsina Moghimnejadhosseini, Farzan Fahim, Daria Beliuk, Guive Sharifi, Alireza Zali

**Affiliations:** 1 Faculty of Medicine, Semmelweis University, Budapest, Hungary; 2 Center of Neurosurgery and Neurointervention, Semmelweis University, Budapest, Hungary; 3 Heart and Vascular Center, Semmelweis University, Budapest, Hungary; 4 Center of Psychology and Psychotherapy, Semmelweis University, Budapest, Hungary; 5 Department of Neurosurgery, Shohada-E Tajrish Hospital, Shahid Beheshti University of Medical Sciences, Tehran, Iran; 6 Functional Neurosurgery Research Center, Research Institute of Functional Neurosurgery, Shohada Tajrish Comprehensive Neurosurgical Center, Tehran, Iran; 7 Loghman Hospital, Shahid Beheshti University of Medical Sciences, Tehran, Iran; 8 Skull Base Division, Shahid Beheshti University of Medical Sciences, Tehran, Iran

**Keywords:** Drug-resistant epilepsy, Epilepsy surgery, Radiofrequency thermocoagulation, Seizure outcome, Stereoelectroencephalography, Resective surgery, Epileptogenic network

## Abstract

Stereoelectroencephalography-guided radiofrequency thermocoagulation (SEEG-RFTC) is used not only as a minimally invasive treatment for drug-resistant focal epilepsy, but also as an in vivo test of an SEEG-defined epileptogenic network. Whether response to RFTC is associated with the outcome of subsequent surgery remains uncertain. We evaluated seizure outcomes after later resective, disconnective, or ablative surgery and the association between RFTC response and later surgical outcome. PubMed/MEDLINE, Embase, Scopus, and Web of Science were searched from inception to June 1, 2026. Eligible studies included patients treated with SEEG-RFTC and reporting therapeutic, safety, downstream-treatment, or postsurgical outcomes. Single-arm outcomes were pooled using random-effects logit models. Sparse permanent-complication data were analyzed with a random-intercept logistic model, and responder-versus-nonresponder comparisons with random-effects odds ratios; Hartung–Knapp inference was examined as a small-sample sensitivity analysis. Risk of bias was assessed using Joanna Briggs Institute tools, with additional Quality In Prognosis Studies assessment of the three studies contributing to the prognostic meta-analysis. Twenty-nine original clinical studies met the eligibility criteria. Six studies comprising 194 patients reported extractable outcomes after qualifying subsequent resective, disconnective, or ablative epilepsy surgery; 141 patients achieved a favorable seizure outcome. The pooled favorable-outcome proportion was 72.4% (95% confidence interval [CI], 65.6%–78.3%; *I*^2^ = 0%). Three studies directly compared responders with nonresponders. Responders had numerically higher odds of favorable later outcome under the conventional random-effects model (odds ratio, 3.86; 95% CI, 0.80–18.57; *I*^2^ = 47.1%), but Hartung–Knapp small-sample inference substantially widened the uncertainty (odds ratio, 3.86; 95% CI, 0.19–77.91; *P* = .193). The pooled proportion proceeding to another epilepsy procedure was 22.0% (95% CI, 11.1%–38.8%), with substantial heterogeneity across clinical pathways (*I*^2^ = 84.1%), while permanent neurological complications occurred in 2.7% (95% CI, 1.0%–6.9%). Approximately seven of 10 selected patients had a favorable seizure outcome after subsequent resective, disconnective, or ablative epilepsy surgery following SEEG-RFTC. A positive RFTC response may strengthen confidence in the surgical hypothesis, but current evidence does not establish independent prognostic value. Current evidence does not support using RFTC nonresponse alone to exclude an otherwise appropriate candidate from definitive epilepsy surgery. SEEG-RFTC may therefore serve as a decision-informing component of network-based surgical evaluation rather than solely as a focal ablative treatment.

**Supplementary Information:**

The online version contains supplementary material available at https://doi.org/10.1007/s10143-026-04561-x.

## Key points

Approximately 72% of selected patients had a favorable seizure outcome after qualifying resective, disconnective, or ablative surgery following SEEG-RFTC.

RFTC responders had numerically higher odds of favorable later surgical outcome, but the pooled association was imprecise and sensitive to individual studies.

RFTC response may support multidisciplinary surgical decision-making but should not independently determine candidacy for definitive epilepsy surgery.

**Supplementary Information:**

The online version contains supplementary material available at https://doi.org/10.1007/s10143-026-04561-x.

## Introduction

Drug-resistant focal epilepsy remains a major cause of neurological disability, with persistent seizures contributing to injury, cognitive and psychiatric comorbidity, impaired quality of life, and premature mortality. Resective or ablative surgery offers the greatest likelihood of durable seizure control, but its success depends on accurate localization of the epileptogenic zone and the ability to treat it without unacceptable functional morbidity [1, 2]. This challenge is particularly pronounced in MRI-negative epilepsy, deep or multilobar epileptogenic networks, and cases in which the presumed seizure-onset zone overlaps eloquent cortex [3, 4]. In these settings, stereoelectroencephalography (SEEG) enables three-dimensional characterization of seizure onset, early propagation, and network organization and may refine the boundaries of tissue considered for intervention [5, 6].

SEEG-guided radiofrequency thermocoagulation (RFTC) extends invasive evaluation into a minimally invasive therapeutic procedure [5, 7]. Radiofrequency energy delivered between selected intracerebral electrode contacts produces focal thermolesions within the SEEG-defined seizure-onset zone, early propagation pathways, or strategically selected network nodes, without requiring an additional craniotomy [7, 8]. Depending on the underlying substrate and surgical constraints, RFTC may serve as definitive treatment, palliation when conventional surgery is unsafe, or an intermediate step before resection, disconnection, or laser ablation [9–11]. Its limited lesion volume, however, may be insufficient for extensive or distributed epileptogenic networks, particularly when only part of the seizure-onset network can be safely treated [12].

Previous evidence syntheses have focused primarily on seizure freedom, responder rates, and procedural morbidity after RFTC itself, demonstrating clinically meaningful benefit but substantial heterogeneity in patient selection, anatomical substrate, targeting strategy, and outcome definition [13–16]. Considerably less attention has been given to the role of RFTC within the broader epilepsy surgery pathway, including its potential value in informing later definitive treatment [3, 9]. A clinical response to RFTC may function as an in vivo perturbation test: transient or sustained seizure improvement could support the causal relevance of the targeted network and increase confidence in a subsequent resective, disconnective, or ablative strategy [17, 18]. Several cohorts have suggested such a relationship, but the available evidence remains dispersed, methodologically heterogeneous, and derived largely from small observational samples [19, 20].

We therefore conducted a systematic review and meta-analysis to estimate seizure outcomes after subsequent resective, disconnective, or ablative epilepsy surgery following SEEG-guided RFTC and to assess whether clinical response to RFTC was associated with favorable later surgical outcome. Secondary objectives were to quantify progression to subsequent surgery, summarize therapeutic response and seizure freedom after RFTC, characterize complications, and explore clinical and methodological sources of heterogeneity.

## Methods

### Protocol, registration, and reporting

**Fig. 1:**
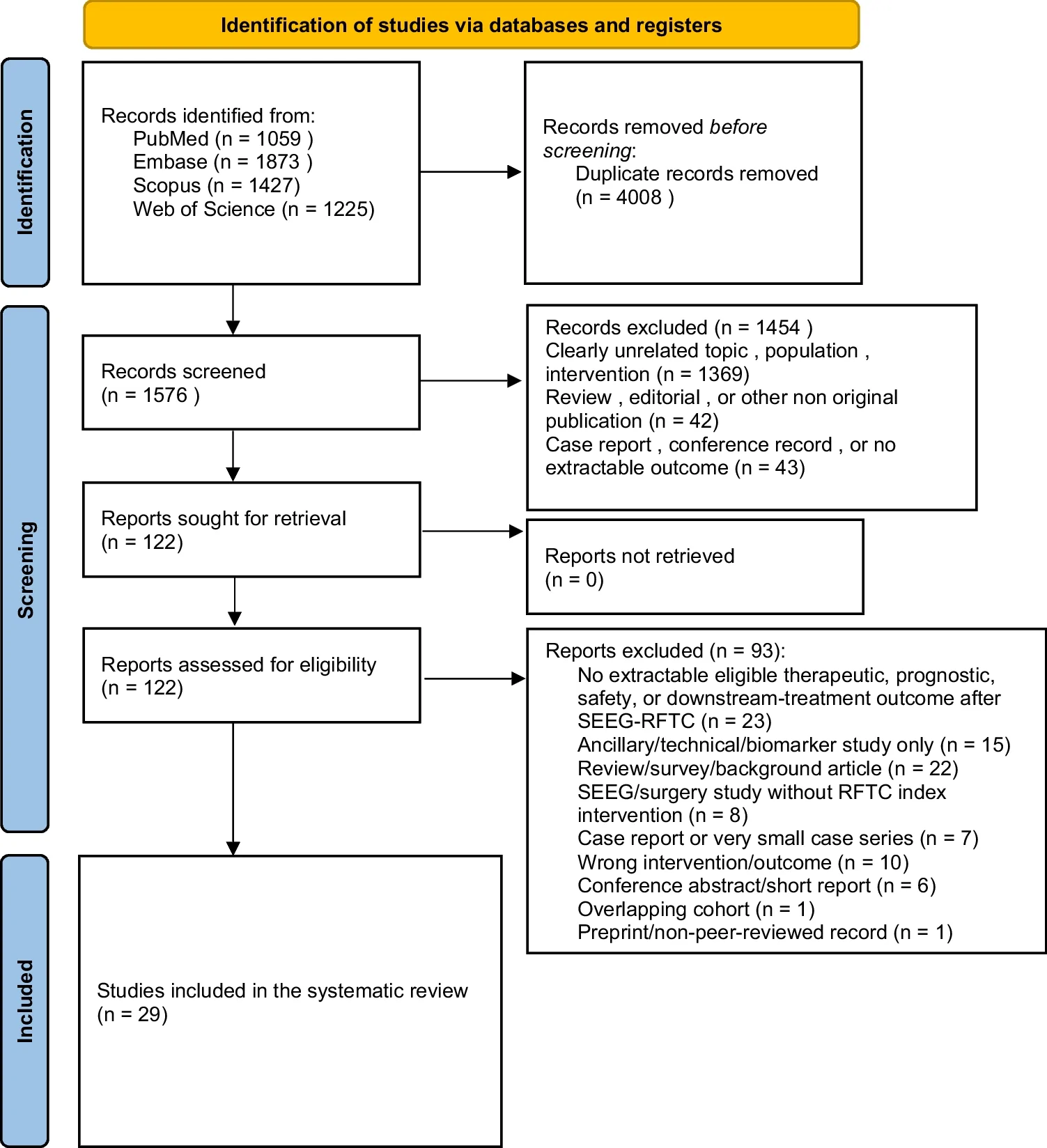
PRISMA 2020 flow diagram of study identification and selection. The database searches identified 5584 records, of which 4008 were removed as duplicates. After screening 1576 unique records, 122 reports underwent full-text assessment and 93 were excluded. Twenty-nine original clinical studies met the eligibility criteria and were included in the systematic review. PRISMA, Preferred Reporting Items for Systematic Reviews and Meta-Analyses; RFTC, radiofrequency thermocoagulation; SEEG, stereoelectroencephalography

This systematic review and meta-analysis was conducted according to a prespecified protocol registered in the International Prospective Register of Systematic Reviews (PROSPERO; CRD420261430081). Reporting followed the Preferred Reporting Items for Systematic Reviews and Meta-Analyses 2020 statement [21]. The completed PRISMA 2020 checklist is provided in Supplementary File 1, and the study-selection process is presented in the PRISMA flow diagram (Fig. 1).

### Information sources and search strategy

PubMed/MEDLINE, Embase, Scopus, and Web of Science Core Collection were searched from database inception to June 1, 2026. No publication-date or language restrictions were applied. Potentially eligible non-English reports were translated into English for eligibility assessment and data extraction, and relevant clinical and numerical information was verified against the original publication.

The search combined controlled vocabulary and free-text terms representing three core concepts: epilepsy; stereoelectroencephalography or intracranial depth-electrode monitoring; and radiofrequency thermocoagulation or ablation. Common terms included *epilepsy*, *seizure*, *stereoelectroencephalography*, *stereo-EEG*, *SEEG*, *depth electrode*, *intracranial EEG*, *radiofrequency*, *thermocoagulation*, *thermoablation*, *ablation*, and *RFTC*, together with related spelling variants. Medical Subject Headings and Emtree terms were incorporated where available, and the syntax was adapted to the indexing structure and field codes of each database.

Complete reproducible strategies for all databases, including database-specific subject headings, field tags, Boolean operators, search dates, and retrieval totals, are provided in Supplementary File 2. Reference lists of eligible original studies and relevant evidence syntheses were also examined to identify potentially missed reports and publications describing overlapping cohorts.

### Eligibility criteria

Eligibility was defined according to a prespecified population, intervention, comparator, outcomes, and study-design framework, summarized in Supplementary Table S1.

#### Population

Eligible original studies included children, adults, or mixed-age populations with drug-resistant focal epilepsy undergoing SEEG-guided radiofrequency thermocoagulation. Temporal, extratemporal, lesional, MRI-negative, multifocal, and network-based epilepsies were eligible. Relevant etiological and anatomical substrates included focal cortical dysplasia, periventricular nodular heterotopia, hypothalamic hamartoma, mesial temporal abnormalities, and epileptogenic networks involving deep-seated or eloquent structures.

Studies containing mixed populations were eligible only when data relating to patients treated with SEEG-guided RFTC could be extracted separately or reliably attributed to the eligible population.

#### Intervention

The index intervention was radiofrequency thermocoagulation or radiofrequency ablation delivered through stereotactically implanted intracerebral electrodes used for SEEG evaluation. Alternative terminology was accepted when the procedural description established that thermocoagulation had been performed through SEEG depth-electrode contacts. Studies in which RFTC was performed using a separate stereotactic lesioning approach, or in which thermocoagulation through SEEG depth-electrode contacts could not be established from the procedural description, were excluded.

For the principal downstream-surgery analysis, eligible subsequent procedures included lesionectomy, tailored cortical resection, temporal resection, selective amygdalohippocampectomy, disconnective surgery, laser interstitial thermal therapy, or another definitive stereotactic ablative procedure performed after RFTC. Neuromodulatory procedures were documented as part of the subsequent treatment pathway but were not combined with resective, disconnective, or ablative procedures in the strict primary outcome analysis.

#### Comparator

A comparator was not required for single-arm outcome analyses. For the principal prognostic analysis, the comparison was between patients who experienced a clinical response after RFTC and those who did not. RFTC response was preferentially defined as a reduction of at least 50% in seizure frequency or temporary or sustained seizure freedom. Study-specific definitions were accepted when clearly reported and were recorded during data extraction.

#### Outcomes

The primary outcome was favorable seizure outcome after subsequent resective, disconnective, or ablative epilepsy surgery following SEEG-guided RFTC. Favorable outcome was preferentially defined as Engel classes I–II or ILAE classes 1–3. When these classifications were unavailable, a clearly defined and clinically comparable author-reported outcome was accepted. The longest available postoperative follow-up was used when several assessment timepoints were reported.

The principal prognostic outcome was the association between clinical response to RFTC and favorable seizure outcome after subsequent surgery. Secondary outcomes were the proportion of patients proceeding to a subsequent epilepsy procedure, clinically meaningful seizure reduction after RFTC, seizure freedom after RFTC, duration of therapeutic benefit after RFTC, and permanent neurological complications attributable to SEEG-guided RFTC.

#### Study designs and exclusion criteria

Eligible original evidence comprised nonrandomized prospective or retrospective cohort studies, comparative observational studies, and case series reporting extractable cohort-level clinical outcomes.

Studies were excluded when they did not involve an eligible drug-resistant epilepsy population; did not evaluate SEEG-guided RFTC as the index intervention; did not report an eligible therapeutic, prognostic, safety, or downstream-treatment outcome; contained mixed interventions without separately extractable RFTC data; or contained duplicate or overlapping data already represented by a more complete report. Case reports, very small case series without analytically usable cohort-level data, animal or laboratory studies, protocols, editorials, correspondence without original clinical data, and purely technical studies without eligible patient outcomes were also excluded.

Systematic reviews, meta-analyses, narrative reviews, technical syntheses, and abstract-only reports were not eligible as included studies. Relevant publications were used only for citation searching and contextual interpretation and contributed no independent patient observations or effect estimates to quantitative synthesis. The complete eligibility framework and standardized full-text exclusion criteria are presented in Supplementary Tables S1 and S2.

### Study selection

Records retrieved from all databases were exported to EndNote 2025 (Clarivate, Philadelphia, PA, USA). Duplicate citations were identified using automated bibliographic matching and subsequently verified manually by comparing titles, authors, publication years, journal details, and digital object identifiers.

A standardized title and abstract screening form was developed by AMM. AMM and DB independently screened every title and abstract against the prespecified eligibility criteria. Each reviewer classified records as eligible, ineligible, or uncertain without access to the other reviewer’s decision. Disagreements were resolved through discussion and, when consensus could not be reached, adjudication by FF.

Reports retained after title and abstract screening underwent independent full-text assessment by DB and SMN using standardized inclusion and exclusion worksheets developed by AMM. Both reviewers evaluated every full-text report independently and recorded the eligibility decision and, where applicable, a prespecified reason for exclusion. Disagreements were resolved through discussion and adjudication by AMM.

No record was excluded solely through an automated screening decision. When several reports originated from the same institution or appeared to describe overlapping populations, recruitment periods, centers, participant characteristics, and intervention details were compared. The most complete report was used for each relevant outcome, and the same participants were not counted more than once within an individual meta-analysis. The complete screening process is shown in Fig. 1, and full-text exclusion decisions are provided in Supplementary Table S2.

### Data extraction

A standardized data-extraction form was developed and piloted by AMM. Before formal extraction, DB and FF were trained in the eligibility criteria, variable definitions, outcome hierarchy, handling of missing information, identification of overlapping cohorts, and reconstruction of prognostic contingency tables.

DB and FF independently extracted data from every eligible record. The completed datasets were compared systematically, and discrepancies were resolved through re-examination of the source publication and discussion between the reviewers. Unresolved discrepancies were adjudicated by AMM.

Extracted study characteristics included first author, publication year, country, institution, study design, enrollment period, prospective or retrospective data collection, cohort size, funding, conflicts of interest, and possible cohort overlap. Participant characteristics included age, sex, epilepsy duration, epilepsy localization, MRI status, etiology or pathology, previous epilepsy surgery, and baseline seizure burden.

Procedural characteristics included the indication for SEEG, RFTC anatomical targets, seizure-onset and early-propagation contacts, targeting strategy, technical parameters, number of treated contacts or thermolesions, repeated or staged treatment, and procedural complications.

Outcome data included the definition and timing of clinical response after RFTC, numbers of responders and nonresponders, seizure freedom, duration of benefit, number proceeding to subsequent treatment, subsequent procedure type, interval between RFTC and later surgery, postoperative follow-up, Engel or ILAE outcome, and permanent neurological complications.

For the prognostic analysis, four-cell data were extracted whenever available: RFTC responders with favorable subsequent-surgery outcomes, RFTC responders with unfavorable outcomes, RFTC nonresponders with favorable outcomes, and RFTC nonresponders with unfavorable outcomes. Reported adjusted effect estimates were recorded separately. Information not reported by the original investigators was coded as not reported and was not interpreted as an absent or zero event.

Core characteristics of the included original studies are summarized in Table 1. The complete extraction dataset and study-role classifications are provided in Supplementary File 3.

**Table 1:** Characteristics, treatment pathways, and outcomes of the 29 included original clinical studies

Study	Country	Design/article type	Population/epilepsy phenotype	Age group	RFTC cohort, n	RFTC outcome and follow-up	Subsequent intervention and outcome	Evidence role
Bourdillon 2017	France	Prospectively followed retrospective single-center cohort	Mixed-age drug-resistant focal epilepsy	Mixed	162	Responder 108/162 at 2 mo and 78/162 at 12 mo; seizure-free 41/162 at 2 mo and 11/162 at 12 mo	91 conventional surgeries; 67/91 Engel I–II	Primary + prognostic quantitative
Chipaux 2019	France; United Kingdom	Multicenter pediatric cohort	Pediatric drug-resistant focal epilepsy, mainly malformations of cortical development	Pediatric	46	Responder 32/46 and seizure-free 20/46 at 1 mo	30 resections; 24/30 Engel I–II	Primary + prognostic quantitative
Panadés-De Oliveira 2023	Spain	Single-center retrospective observational cohort	MRI-negative drug-resistant focal epilepsy	Adult	31 MRI-negative patients (55 total RFTC)	Responder 14/31; seizure-free 8/31; mean FU 30.9 mo	10 resection/LITT procedures; 9 with final outcome: 9/9 Engel I–II and 8/9 Engel I	Prognostic quantitative
Shields 2023	United States	Single-center retrospective cohort	Drug-resistant focal epilepsy, lesional and nonlesional	Mixed	57	Seizure-free after RFA alone 12/49 with delayed follow-up; median overall FU 39.1 mo	32 delayed procedures (26 LITT, 5 resection, 1 RNS); 24/32 Engel I–II overall; favorable outcomes for the 31 qualifying LITT/resection procedures were not separately reported	Narrative original
Du 2024	China	Single-center retrospective cohort	Drug-resistant focal epilepsy, lesional and nonlesional	Mixed	40 analyzed (43 treated)	RFTC-alone seizure-free 8/40; mean FU 28.8 mo	24 delayed excision surgeries; 16/24 Engel I–II	Primary quantitative
Dimova 2017	France	Single-center retrospective cohort	Complex drug-resistant focal epilepsy	Mixed	23	Responder 8/23; sustained seizure freedom 1/23; mean FU 32.1 mo	9 resections; 4/9 Engel I	Primary quantitative
Mirandola 2017	Italy	Single-center retrospective cohort	Periventricular nodular heterotopia-related drug-resistant epilepsy	Mixed	17	Seizure-free 13/17 after SEEG-RFTC; overall mean FU 50 mo	4 resections after RFTC failure; 3/4 Engel I	Secondary quantitative
Moles 2018	France	Retrospective comparative cohort	SEEG-confirmed drug-resistant temporal lobe epilepsy	Mixed	21	Responder 10/21; seizure-free 0/21 at 12 mo	19 anterior temporal lobectomies; 14/19 Engel Ia	Primary quantitative
Contento 2021	France	Retrospective biomarker cohort	Focal drug-resistant epilepsy	Mixed	38	Responder 19/38; seizure-free 8/38; median FU 39 mo	21 resections; 16/21 favorable surgical outcome	Primary quantitative
Huang 2024	China	Single-center retrospective cohort	MRI-negative drug-resistant focal epilepsy	Mixed	30	Seizure-free 11/30; median FU 30 mo	12 subsequent procedures (11 open surgery, 1 VNS); postoperative outcome not fully extractable	Secondary quantitative
Vasquez 2025	United States	Retrospective single-institution cohort	Drug-resistant focal epilepsy	Mixed	25	Responder 12/25; seizure-free 3/25; median FU 7.2 mo	20 further interventions; outcome available in 17: 13/17 Engel I–III (12/17 Engel I–II)	Secondary quantitative
Chandra 2025	India	Retrospective single-center observational cohort	Drug-resistant focal epilepsy and epileptogenic networks	Mixed	61	≥ 50% early reduction 58/61; 44 had sustained transient improvement; ≥ 12-mo outcome available	29 downstream interventions (27 resection/disconnection procedures and 2 VNS procedures); 18/29 ILAE 1–2 overall; favorable outcomes were not separately reported for the 27 qualifying resective/disconnective procedures	Secondary quantitative
Catenoix 2015	France	Single-center retrospective cohort	Malformation of cortical development-related focal epilepsy	Mixed	14	Responder 9/14; long-lasting seizure freedom 6/14; median FU 41.7 mo	No later surgery reported	Secondary quantitative
Cossu 2015	Italy	Single-center retrospective cohort	Complex drug-resistant focal epilepsy	Mixed	89	Responder 25/89; seizure-free 16/89	51 microsurgical resections; RFTC-specific postoperative outcome not extractable	Secondary quantitative
Méreaux 2020	France	Retrospective consecutive case series	Drug-resistant epilepsy explored with SEEG	Mixed	8 RFTC patients	Responder 7/8; seizure-free 3/8	33 resections in the overall SEEG cohort; RFTC-specific downstream outcome not reported	Secondary quantitative
Liu 2021	China	Retrospective consecutive cohort	Hypothalamic hamartoma-related drug-resistant epilepsy	Mixed	27	Responder 23/27; seizure-free 19/27; mean FU 27.3 mo	No later surgery reported	Secondary quantitative
Xu 2022	China	Retrospective consecutive case series	Drug-resistant focal epilepsy involving eloquent motor cortex	Mixed	15	Responder 12/15; seizure-free 9/15; mean FU 22.1 mo	1 anterior thalamic DBS; outcome not reported	Secondary quantitative
Li 2023 (PET/MRI)	China	Single-center retrospective cohort	Predominantly MRI-negative drug-resistant epilepsy	Mixed	27	Responder 23/27 using Engel I–III; seizure-free 15/27; mean FU 27.8 mo	No later surgery reported	Secondary quantitative
Jean 2024	China	Single-center retrospective cohort	Unilateral mesial temporal lobe epilepsy	Mixed/unclear	22	Responder and seizure-free 16/22; mean FU 24.2 mo	No later surgery reported	Secondary quantitative
Kreinter 2024	Canada	Single-center retrospective cohort	Adult focal drug-resistant epilepsy	Adult	18	Responder 12/18 at 1 mo, 10 at 6 mo, and 7 at 12 mo; seizure-free 9, 3, and 2, respectively	6 resections; postoperative outcome not reported	Secondary quantitative
Li 2024	China	Single-center retrospective observational cohort	Mesial temporal lobe epilepsy with hippocampal sclerosis	Mixed	35	Responder 22/35; seizure-free 12 at 60 mo; median FU 77 mo	2 later procedures (1 VNS, 1 anterior temporal lobectomy); outcomes not reported	Secondary quantitative
Liu 2024	China	Single-center retrospective observational cohort	Refractory focal epilepsy, heterogeneous etiologies	Mixed	121	Responder 82/121; seizure-free 54/121; mean FU 18.7 mo	39 resection or neuromodulation procedures; outcomes not reported	Secondary quantitative
Slegers 2024	Netherlands	Prospectively collected retrospective cohort	Periventricular nodular heterotopia-related focal epilepsy	Adult	24	Responder 17/24; seizure-free 11/24; median FU 35 mo	No later surgery reported	Secondary quantitative
Chen 2025	China	Retrospective observational cohort	Pediatric MRI-negative drug-resistant focal epilepsy	Pediatric	33	Responder 25/33; seizure-free 17/33; mean FU 27.2 mo	No later surgery reported	Secondary quantitative
Liu 2025	China	Retrospective single-center cohort	Hypothalamic hamartoma-related drug-resistant epilepsy	Mixed	30	ILAE 1–2 in 20/30 after last RFTC; mean FU 66.8 mo after last RFTC	6 repeat RFTC procedures; no later resection/LITT reported	Narrative original
Yan 2025	China	Single-center observational biomarker cohort	Drug-resistant focal epilepsy, heterogeneous etiologies	Mixed	44	Clinical improvement 25/44; median FU 32 mo	16 subsequent surgical treatments; types/outcomes not reported	Secondary quantitative
Chen 2026	China	Multicenter retrospective real-world cohort	Pediatric focal drug-resistant epilepsy	Pediatric	111	Responder and seizure-free 73/111; mean FU 30.7 mo	No later surgery reported	Secondary quantitative
Shen 2026	China	Retrospective cohort with machine-learning analysis	Focal cortical dysplasia-related drug-resistant epilepsy	Mixed	31	Responder and seizure-free 17/31; mean FU 25.5 mo	10 subsequent resections; outcomes not reported	Secondary quantitative
Xu 2026	China	Multicenter retrospective cohort	Drug-resistant epilepsy, heterogeneous etiologies	Mixed	63	Responder and seizure-free 41/63; mean FU 23.0 mo	No later surgery reported	Secondary quantitative

Data are presented for the SEEG-guided RFTC cohort unless otherwise indicated. RFTC response was defined according to each original report, most commonly as a reduction of ≥ 50% in seizure frequency. Favorable outcome after subsequent surgery was defined according to each original study, most commonly as Engel class I–II or ILAE class 1–3. The table includes only the 29 original clinical studies meeting the eligibility criteria. Contextual and citation-searching publications were not included in the evidence base and contributed no independent patient observations or effect estimates. Abbreviations: *DBS* deep brain stimulation; *FU* follow-up; *ILAE* International League Against Epilepsy; *LITT* laser interstitial thermal therapy; *MRI* magnetic resonance imaging; *RFA* radiofrequency ablation; *RFTC* radiofrequency thermocoagulation; *RNS* responsive neurostimulation; *SEEG* stereoelectroencephalography; *VNS* vagus nerve stimulation

### Risk-of-bias assessment

DB and SMN independently assessed risk of bias for the 29 included original clinical studies using the Joanna Briggs Institute critical appraisal checklist appropriate to each study design. Case series were evaluated using the JBI Critical Appraisal Checklist for Case Series, and observational cohort studies were evaluated using the JBI Critical Appraisal Checklist for Cohort Studies. Each applicable item was classified as yes, no, unclear, or not applicable. Disagreements were resolved through discussion and, where necessary, adjudication by AMM.

JBI judgments were evaluated at the item level and were not converted to numerical scores or overall low-, moderate-, or high-risk classifications. Contextual reviews and other publications that did not constitute included original clinical studies were not subjected to risk-of-bias assessment for the present review.

**Fig. 2:**
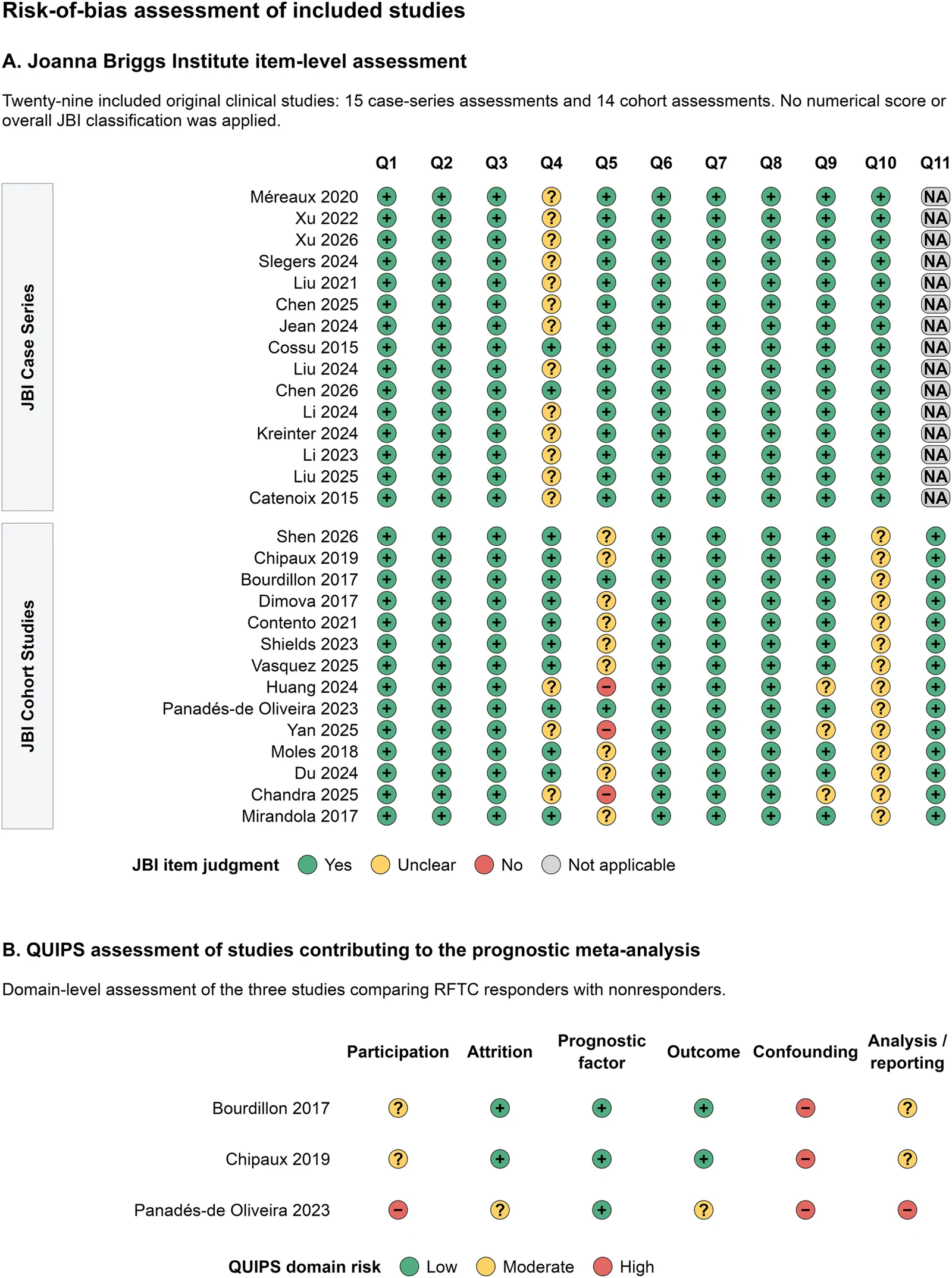
Risk-of-bias assessment of the included original clinical studies and prognostic evidence. Panel **A** shows item-level judgments for the 29 included original clinical studies using the Joanna Briggs Institute critical appraisal checklist appropriate to each study design. Fifteen studies were assessed using the JBI Critical Appraisal Checklist for Case Series and 14 using the JBI Critical Appraisal Checklist for Cohort Studies. Each applicable item was rated as yes, no, unclear, or not applicable; no numerical score or overall JBI risk category was assigned. Panel **B** shows domain-level Quality In Prognosis Studies (QUIPS) assessments for the three studies contributing to the responder-versus-nonresponder prognostic meta-analysis. QUIPS domains were study participation, study attrition, prognostic-factor measurement, outcome measurement, study confounding, and statistical analysis and reporting, each judged as low, moderate, or high risk of bias. JBI, Joanna Briggs Institute; QUIPS, Quality In Prognosis Studies

Because the responder-versus-nonresponder meta-analysis addressed a prognostic-factor question, the three studies contributing to that analysis were additionally assessed using the Quality In Prognosis Studies (QUIPS) framework. The six QUIPS domains were study participation, study attrition, prognostic-factor measurement, outcome measurement, study confounding, and statistical analysis and reporting. Each domain was judged as having low, moderate, or high risk of bias. Item-level JBI judgments and domain-level QUIPS assessments are displayed in Fig. 2 and provided in Supplementary File 4.

### Data synthesis and statistical analysis

Eligible original studies were synthesized narratively according to population characteristics, epilepsy substrate, RFTC technique, therapeutic outcomes, subsequent treatment pathway, and analytical role. Contextual reviews informed interpretation and citation searching but did not contribute patient observations or independent effect estimates.

Quantitative synthesis was undertaken when at least two clinically compatible original studies reported an extractable numerator and denominator or a comparable effect estimate. Separate meta-analyses were performed for favorable seizure outcome after subsequent epilepsy surgery, progression to a subsequent epilepsy procedure, clinically meaningful seizure reduction after RFTC, seizure freedom after RFTC, the association between RFTC response and later surgical outcome, and permanent neurological complications.

#### Meta-analysis of single-arm proportions

For each study *i*, the observed proportion was calculated as:

$${p}_{i}=\frac{{x}_{i}}{{n}_{i}}$$

where ${x}_{i}$ was the number of participants experiencing the outcome and ${n}_{i}$ was the corresponding eligible denominator.

For the favorable later-surgery outcome, progression to subsequent intervention, clinically meaningful RFTC response, and seizure-freedom outcomes, proportions were transformed to the logit scale:

$${y}_{i}=\mathrm{log}\left(\frac{{p}_{i}}{1-{p}_{i}}\right)$$

The within-study variance was estimated as:

$${v}_{i}=\frac{1}{{x}_{i}}+\frac{1}{{n}_{i}-{x}_{i}}$$

where estimable. For studies reporting no events or events in all participants, a continuity correction was applied only when required to permit logit transformation.

Random-effects models were used because clinical and methodological heterogeneity was anticipated across study populations, epilepsy substrates, targeting strategies, outcome definitions, subsequent interventions, and follow-up durations. Between-study variance, ${\tau }^{2}$, was estimated using restricted maximum likelihood. Random-effects weights were calculated as:

$${w}_{i}=\frac{1}{{v}_{i}+{\uptau }^{2}}$$

The pooled logit estimate was calculated as:

$$\widehat{\upmu }=\frac{{\sum}_{i=1}^{k}{w}_{i}{y}_{i}}{{\sum}_{i=1}^{k}{w}_{i}}$$

The pooled estimate was then back-transformed to the proportion scale:

$$\widehat{p}=\frac{\mathrm{exp}\left(\widehat{\upmu }\right)}{1+\mathrm{exp}\left(\widehat{\upmu }\right)}$$

Pooled proportions were reported with 95% confidence intervals.

#### Prognostic meta-analysis

The principal prognostic analysis compared the odds of favorable subsequent-surgery outcome between patients who responded clinically to RFTC and those who did not.

For each study with an extractable 2 × 2 contingency table, the study-specific odds ratio was calculated as:

$$O{R}_{i}=\frac{{a}_{i}{d}_{i}}{{b}_{i}{c}_{i}}$$

where ${a}_{i}$ represented responders with favorable outcomes, ${b}_{i}$ responders with unfavorable outcomes, ${c}_{i}$ nonresponders with favorable outcomes, and ${d}_{i}$ nonresponders with unfavorable outcomes.

The sampling variance of the log odds ratio was estimated as:

$$Var\left[\mathrm{log}\left(O{R}_{i}\right)\right]=\frac{1}{{a}_{i}}+\frac{1}{{b}_{i}}+\frac{1}{{c}_{i}}+\frac{1}{{d}_{i}}$$

A continuity correction of 0.5 was applied to all four cells only when a zero cell prevented estimation. Study-specific log odds ratios were pooled using an inverse-variance random-effects model, with ${\tau }^{2}$ estimated using restricted maximum likelihood. The pooled log odds ratio was exponentiated for presentation:

$$O{R}_{pooled}=\mathrm{exp}\left(\widehat{\uptheta }\right),$$

where $\widehat{\theta }$ represented the pooled log odds ratio. An odds ratio greater than 1 indicated higher odds of a favorable subsequent-surgery outcome among RFTC responders. Because only three studies contributed to the responder-versus-nonresponder analysis, a Hartung–Knapp adjustment was applied as a small-sample sensitivity analysis using the same restricted maximum-likelihood estimate of between-study variance and t-based inference with k − 1 degrees of freedom.

#### Permanent neurological complications

Permanent neurological complications were analyzed separately because events were sparse and several studies reported no permanent complications. The strict safety analysis included only nonoverlapping original cohorts with a clearly reported RFTC denominator and permanent neurological complications attributable to SEEG-guided RFTC.

A random-intercept logistic generalized linear mixed model was fitted using the binomial likelihood:

$${X}_{i}\sim Binomial\left({n}_{i},{p}_{i}\right)$$

with:

$$logit\left({p}_{i}\right)=\mathrm{log}\left(\frac{{p}_{i}}{1-{p}_{i}}\right)=\upmu +{u}_{i}$$

where:

$${u}_{i}\sim N\left(0,{\uptau }^{2}\right)$$

The pooled permanent-complication proportion was obtained by back-transforming the estimated intercept:

$$\widehat{p}=\frac{\mathrm{exp}\left(\widehat{\upmu }\right)}{1+\mathrm{exp}\left(\widehat{\upmu }\right)}$$

Between-study variance for this model was estimated using maximum likelihood. Because the model used the binomial likelihood directly, no continuity correction was required.

#### Heterogeneity and sensitivity analyses

Statistical heterogeneity was characterized using Cochran’s*Q*, between-study variance${\tau }^{2}$, and the ${I}^{2}$ statistic. For the calculation of Cochran’s, fixed-effect inverse-variance weights were defined as:

$${w}_{i}^{\left(F\right)}=\frac{1}{{v}_{i}}$$

The corresponding inverse-variance weighted mean was calculated as:

$$\overline{{y }_{F}}=\frac{{\sum}_{i=1}^{k}{w}_{i}^{\left(F\right)}{y}_{i}}{{\sum}_{i=1}^{k}{w}_{i}^{\left(F\right)}}$$

Cochran’s *Q* was calculated as:

$$Q={\sum}_{i=1}^{k}{w}_{i}^{\left(F\right)}{\left({y}_{i}-\overline{{y }_{F}}\right)}^{2}$$

The ${I}^{2}$ statistic was calculated as:

$${I}^{2}=\mathrm{max}\left(0,\frac{Q-\left(k-1\right)}{Q}\right)\times 100\backslash \%$$

Leave-one-out analyses were performed for models containing at least three studies to assess the influence of individual studies on the pooled estimate. In each iteration, one study was removed and the model was refitted using the same statistical specification.

For the primary favorable later-surgery outcome, an additional sensitivity analysis was performed restricting the dataset to studies in which postoperative seizure outcomes were documented at 12 months or longer for the contributing surgical cohort. The sensitivity analysis used the same random-effects logit model and restricted maximum-likelihood estimation as the primary analysis.

Exploratory subgroup analyses and meta-regression were not undertaken when the number of contributing studies or the consistency of reported covariates was insufficient for reliable estimation. Formal tests of funnel-plot asymmetry were not performed for the primary later-surgery, prognostic, or permanent-complication analyses because fewer than 10 studies contributed to each model. For the broader single-arm secondary outcomes that included at least 10 studies, funnel plots were generated and interpreted descriptively; formal asymmetry tests were not conducted because funnel-plot appearance in meta-analyses of proportions may be influenced by substantial clinical heterogeneity, variation in outcome definitions and follow-up, and proportions approaching the boundaries of zero or one.

All analyses were performed using R version 4.6.0 (R Foundation for Statistical Computing, Vienna, Austria), principally with the meta and metafor packages [22, 23]. Forest plots and customized graphical outputs were prepared using ggplot2 where required [24]. Statistical tests were two-sided, with *P* < 0.05 considered statistically significant. Interpretation emphasized effect magnitude, confidence-interval width, consistency, and clinical relevance rather than statistical significance alone.

## Results

### Study selection

The database searches identified 5584 records: 1059 from PubMed/MEDLINE, 1873 from Embase, 1427 from Scopus, and 1225 from Web of Science. After removal of 4008 duplicate records, 1576 unique records underwent title and abstract screening. Of these, 1454 were excluded because they addressed an unrelated population, topic, or intervention (*n* = 1369); represented a review, editorial, or other nonoriginal publication type (*n* = 42); or were a case report, conference record, or report without an extractable outcome (*n* = 43). The complete title and abstract screening dataset is provided in Supplementary File 5.

All 122 reports sought for full-text assessment were retrieved. Ninety-three reports were excluded: 23 did not provide an extractable eligible therapeutic, prognostic, safety, or downstream-treatment outcome after SEEG-guided RFTC; 15 were limited to ancillary, technical, or biomarker outcomes; 22 were reviews, surveys, or background publications; eight evaluated SEEG or epilepsy surgery without RFTC as the index intervention; seven were case reports or very small case series; 10 evaluated an ineligible intervention or outcome; six were conference abstracts or short reports; one represented an overlapping cohort; and one was a non-peer-reviewed preprint. Full-text exclusions and corresponding reasons are detailed in Supplementary Table S2.

Twenty-nine original clinical studies met the eligibility criteria and were included in the systematic review. Relevant reviews, evidence syntheses, and other contextual publications were used only for citation searching and interpretation and were not counted as included evidence. Following re-evaluation of the prespecified intervention definition, three reports using non-SEEG-contact or insufficiently documented depth-electrode RFTC approaches were excluded; none had contributed to any quantitative synthesis. The complete study-selection process is presented in Fig. 1.

### Study and participant characteristics

The 29 included original clinical studies were published between 2015 and 2026 and represented cohorts from Asia (*n* = 15), Europe (*n* = 11), and North America (*n* = 3). Twenty-six were single-center studies and three were multicenter studies. All used observational designs, and most collected data retrospectively. Reported eligible RFTC cohort sizes ranged from 8 to 162 patients, with a median cohort size of 30 among studies reporting a numerical denominator.

Most studies included mixed pediatric and adult populations; three were restricted to pediatric patients and three to adults. Reported study-level mean ages ranged from 8.2 to 33.6 years, although age and epilepsy-duration data were inconsistently reported. Study populations encompassed MRI-negative and structurally lesional drug-resistant focal epilepsy, temporal and extratemporal epilepsy, and epileptogenic networks involving deep-seated or eloquent regions. Frequently represented substrates included focal cortical dysplasia, periventricular nodular heterotopia, hypothalamic hamartoma, hippocampal sclerosis, and other malformations of cortical development [1–3, 5, 8, 9, 19, 25–27].

Subsequent interventions after RFTC included tailored cortical resection, lesionectomy, temporal resection, selective amygdalohippocampectomy, disconnective procedures, laser interstitial thermal therapy, and other definitive stereotactic ablative procedures. Neuromodulatory procedures were reported within some broader treatment-pathway cohorts but were not incorporated into the strict resective, disconnective, or ablative later-surgery outcome analysis. Follow-up duration and the interval between RFTC and subsequent intervention varied considerably across studies. Complete study, participant, procedural, and outcome characteristics are presented in Table 1 and Supplementary File 3.

### Risk of bias

Among the 29 included original clinical studies, 15 were assessed using the JBI Critical Appraisal Checklist for Case Series and 14 using the JBI Critical Appraisal Checklist for Cohort Studies. Risk of bias was evaluated at the item level without numerical scoring or assignment of overall JBI categories. Across the case-series assessments, most checklist items were judged favorably, with the most recurrent uncertainty involving consecutive inclusion. Among cohort studies, the principal concerns involved identification and management of confounding and incomplete or insufficiently described follow-up and statistical reporting. Item-level judgments are shown in Fig. 2A.

The three studies contributing to the responder-versus-nonresponder prognostic meta-analysis were additionally evaluated with QUIPS. Confounding was judged to be at high risk of bias in all three studies because the reported responder–outcome associations were unadjusted and subsequent surgical treatment was clinically selected. Bourdillon et al. and Chipaux et al. were judged to have moderate risk in study participation and statistical analysis/reporting, with low risk in attrition, prognostic-factor measurement, and outcome measurement. Panadés-de Oliveira et al. had high risk in study participation, confounding, and statistical analysis/reporting; moderate risk in attrition and outcome measurement; and low risk in prognostic-factor measurement. The QUIPS assessments are shown in Fig. 2B, with supporting rationales provided in Supplementary File 4.

### Favorable seizure outcome after subsequent surgery

**Fig. 3:**
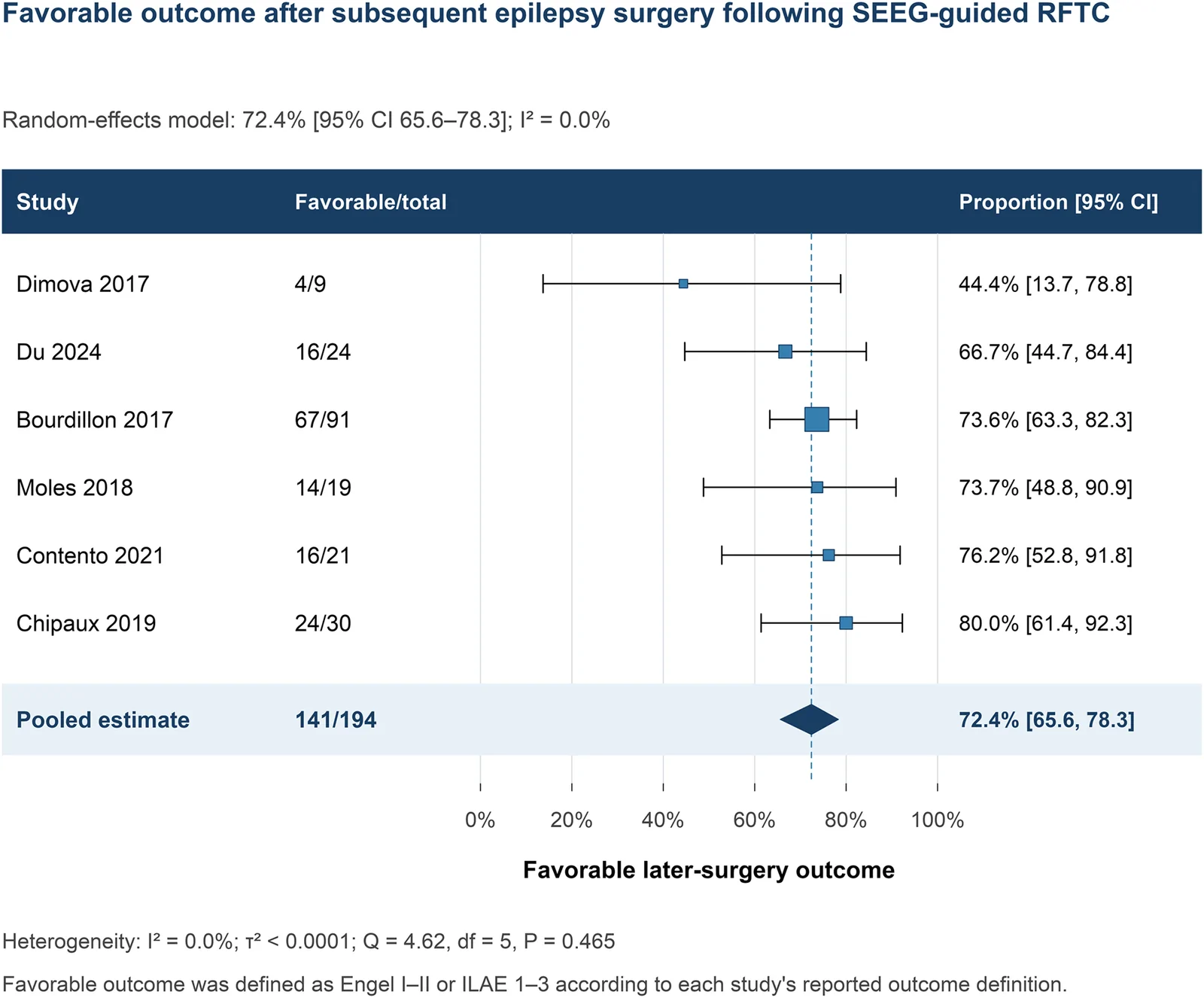
Favorable seizure outcome after subsequent epilepsy surgery following SEEG-guided RFTC. Random-effects meta-analysis of the proportion of patients achieving a favorable seizure outcome after qualifying subsequent resective, disconnective, or ablative epilepsy surgery. Six studies comprising 194 patients contributed to the analysis, and 141 patients achieved a favorable outcome. The pooled favorable-outcome proportion was 72.4% (95% confidence interval, 65.6%–78.3%), with no statistical heterogeneity detected (*I*^2^ = 0%; *τ*^2^ < 0.0001; *Q* = 4.62, df = 5; *P* = 0.465). Squares represent study-specific proportions, with their size proportional to study weight; horizontal lines indicate 95% confidence intervals, and the diamond represents the pooled estimate. CI, confidence interval; RFTC, radiofrequency thermocoagulation; SEEG, stereoelectroencephalography

Six studies comprising 194 patients reported extractable seizure outcomes after qualifying subsequent resective, disconnective, or ablative epilepsy surgery following SEEG-guided RFTC [8, 9, 17, 19, 25, 28]. Favorable outcomes were reported in 141 patients, with study-specific proportions ranging from 44.4% to 80.0%. The random-effects pooled favorable-outcome proportion was 72.4% (95% CI, 65.6%–78.3%), with no statistical heterogeneity detected (*I*^2^ = 0%; *τ*^2^ < 0.0001; *Q* = 4.62, df = 5; *P* = 0.465; Fig. 3). The absence of detected statistical heterogeneity should not be interpreted as evidence of clinical homogeneity, given the small number of contributing cohorts and differences in subsequent procedures, outcome definitions, and follow-up.

Characteristics of the six studies contributing to the primary later-surgery meta-analysis, including the subsequent procedure, favorable-outcome definition, and postoperative follow-up, are summarized in Supplementary Table S3. In a sensitivity analysis restricted to studies in which postoperative seizure outcomes were documented at 12 months or longer for the contributing surgical cohort, four studies comprising 155 patients were eligible, of whom 113 achieved a favorable outcome. The pooled favorable-outcome proportion was 72.8% (95% CI, 65.3%–79.3%), with no statistical heterogeneity detected (*I*^2^ = 0%; *τ*^2^ < 0.0001; *Q* = 0.61, df = 3; *P* = 0.893; Supplementary Figure S1). The estimate was essentially unchanged from the primary analysis.

Chandra et al. [20] reported favorable outcomes in 18 of 29 patients undergoing subsequent intervention; however, this group comprised 27 resective or disconnective procedures and two vagus nerve stimulation procedures, and favorable outcomes were not separately reported for the 27 qualifying procedures. The cohort was therefore excluded from the strict primary later-surgery meta-analysis but retained in the broader downstream-treatment synthesis.

### Association between RFTC response and subsequent-surgery outcome

Three studies provided an extractable comparison of subsequent-surgery outcomes between RFTC responders and nonresponders [3, 9, 25]. Favorable subsequent-surgery outcomes occurred in 59 of 66 RFTC responders and 39 of 64 nonresponders.

**Fig. 4:**
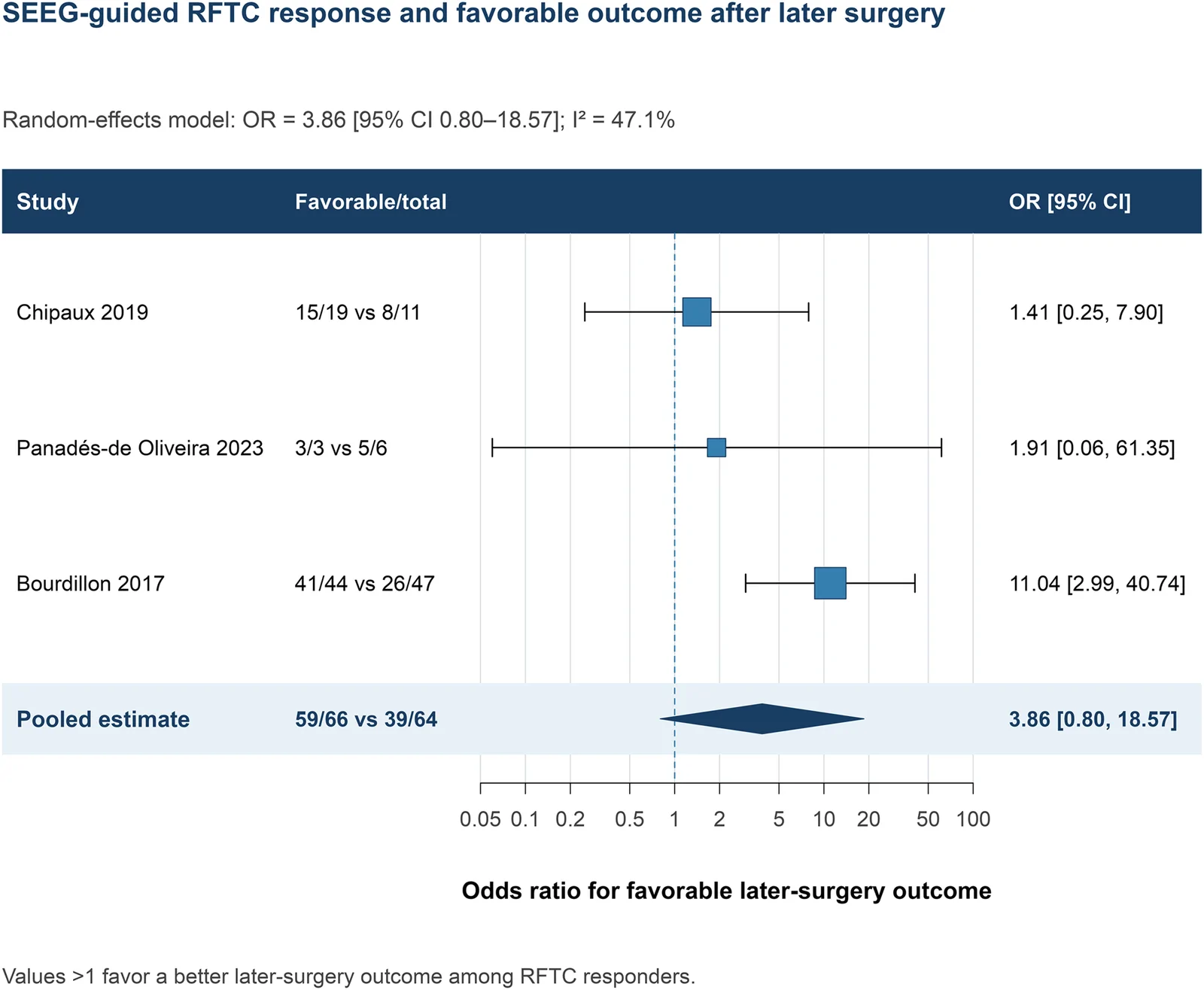
Association between clinical response to SEEG-guided RFTC and favorable outcome after subsequent epilepsy surgery. Random-effects meta-analysis comparing favorable subsequent-surgery outcomes between patients who responded clinically to RFTC and those who did not. Three studies contributed 66 responders and 64 nonresponders. Favorable outcomes occurred in 59 responders and 39 nonresponders. The pooled odds ratio was 3.86 (95% confidence interval, 0.80–18.57; *P* = 0.092), with moderate statistical heterogeneity (*I*^2^ = 47.1%; *τ*^2^ = 0.94). Odds ratios greater than 1 favor RFTC responders. Squares represent study-specific odds ratios, with their size proportional to study weight; horizontal lines indicate 95% confidence intervals, and the diamond represents the pooled estimate. CI, confidence interval; OR, odds ratio; RFTC, radiofrequency thermocoagulation; SEEG, stereoelectroencephalography

The conventional random-effects pooled odds ratio was 3.86 (95% CI, 0.80–18.57; *P* = 0.092), with moderate statistical heterogeneity (*I*^2^ = 47.1%; *τ*^2^ = 0.94; *P* for heterogeneity = 0.151; Fig. 4). The point estimate indicated numerically higher odds of a favorable subsequent-surgery outcome among RFTC responders; however, the confidence interval was wide and included the null. In the Hartung–Knapp small-sample sensitivity analysis, the pooled odds ratio remained 3.86 but the 95% CI widened substantially to 0.19–77.91 (*P* = 0.193). This marked loss of precision reinforces that the responder-versus-nonresponder finding should be interpreted as an exploratory association rather than evidence of independent prognostic value. Study-specific odds ratios ranged from 1.41 to 11.04.

The magnitude and precision of the pooled association varied in leave-one-out analysis. Omission of Chipaux et al. yielded an odds ratio of 8.88 (95% CI, 2.62–30.14), omission of Panadés-de Oliveira et al. yielded an odds ratio of 4.27 (95% CI, 0.57–31.96), and omission of Bourdillon et al. yielded an odds ratio of 1.49 (95% CI, 0.32–7.00). These findings indicated that the prognostic estimate was sensitive to the inclusion of individual studies, particularly the larger Bourdillon cohort. The complete influence analysis is presented in Supplementary Figure S2.

### Progression to subsequent intervention

Twenty-three studies reported the proportion of patients proceeding to a subsequent epilepsy procedure after RFTC. The pooled proportion was 22.0% (95% CI, 11.1%–38.8%), with substantial between-study heterogeneity (*I*^2^ = 84.1%; Supplementary Figure S3).

The intended role of RFTC differed considerably among cohorts. In some studies, RFTC was used primarily as definitive or palliative treatment for patients considered unsuitable for conventional resection, whereas in others it represented an intermediate step within a broader surgical pathway. Follow-up duration, access to definitive surgery, and the types of subsequent procedures included also varied. The pooled estimate therefore reflects the frequency of additional intervention across heterogeneous clinical pathways rather than a uniform probability of proceeding to later surgery.

### Therapeutic outcomes after RFTC

Seventeen studies contributed to the analysis of clinically meaningful seizure reduction after RFTC. The pooled responder proportion was 61.3% (95% CI, 53.0%–69.0%), with substantial statistical heterogeneity (*I*^2^ = 75.2%; Supplementary Figure S4). Clinical response was most frequently defined as a reduction of at least 50% in seizure frequency, although study-specific definitions and assessment timepoints varied.

Seventeen studies also reported seizure freedom after RFTC. The pooled seizure-freedom proportion was 46.8% (95% CI, 36.0%–58.0%), with considerable statistical heterogeneity (*I*^2^ = 82.7%; Supplementary Figure S5). Follow-up ranged from short postprocedural assessments to several years, and some longitudinal cohorts reported decreasing seizure-freedom rates with longer follow-up.

### Duration of RFTC response and other prognostic observations

The duration of benefit after RFTC was not reported consistently enough for quantitative synthesis. In one cohort, patients with favorable outcomes after delayed excision experienced a longer mean period of seizure freedom after RFTC than those with unfavorable later-surgery outcomes (4.9 vs 1.9 months, *P* = 0.007; Du et al., 2024 [19]). Other studies reported temporary seizure improvement or short seizure-free intervals before subsequent treatment, but definitions, assessment schedules, and reported time intervals were heterogeneous.

Electrophysiological, imaging, and technical variables were also evaluated inconsistently. Individual studies examined MRI positivity, the extent and anatomical distribution of thermocoagulation, post-RFTC changes in interictal activity or high-frequency oscillations, and spatial concordance between thermocoagulated and subsequently resected tissue. These variables were not pooled because sufficiently comparable effect estimates were unavailable.

### Safety outcomes

Three independent studies comprising 150 patients reported sufficiently consistent data on permanent neurological complications attributable to SEEG-guided RFTC. Four permanent complications were recorded. Using a random-intercept logistic generalized linear mixed model, the pooled permanent-complication proportion was 2.7% (95% CI, 1.0%–6.9%), with no observed statistical heterogeneity (*I*^2^ = 0%; *τ*^2^ = 0; Wald *χ*^2^ = 0.93; *P* = 0.628; Supplementary Figure S6). The small number of contributing studies and sparse event count limited the precision of the pooled estimate and the assessment of heterogeneity.

Across the broader evidence base, most reported adverse events were transient and included focal motor or sensory deficits, language symptoms, visual disturbance, headache, and procedure-associated seizures. Permanent complications were reported infrequently and consisted mainly of persistent motor, sensory, language, or visual deficits, often following thermocoagulation near eloquent cortex or visual pathways. Studies that did not meet the strict quantitative safety definition were synthesized narratively because of inconsistent definitions, causal attribution, follow-up periods, and reporting denominators. Complete study-level safety data are provided in Supplementary File 3.

#### Additional analyses

The responder-versus-nonresponder association varied substantially according to the study omitted, reflecting the small number of contributing cohorts and the influence of individual studies.

Reliable subgroup analyses according to age group, temporal versus extratemporal localization, MRI status, or subsequent procedure type were not undertaken because subgroup data were sparse and inconsistently reported. Meta-regression was likewise not performed.

Formal tests of funnel-plot asymmetry were not conducted for the favorable later-surgery, prognostic, or permanent-complication analyses because fewer than 10 studies contributed to each model. Funnel plots were generated for clinically meaningful seizure response after RFTC, seizure freedom after RFTC, and progression to a subsequent epilepsy procedure (Supplementary Figures S7–S9, respectively). Some visual asymmetry was present, particularly for seizure freedom and progression to subsequent intervention; however, these findings were interpreted descriptively because substantial clinical and statistical heterogeneity, differences in outcome definitions and follow-up, and boundary-event frequencies limit the ability of funnel plots to distinguish publication bias from other sources of asymmetry.

## Discussion

### Principal findings

This systematic review and meta-analysis evaluated SEEG-guided RFTC not only as a therapeutic intervention but also as a potential decision-informing step before definitive epilepsy surgery. Among selected patients who subsequently underwent qualifying resective, disconnective, or ablative surgery, the pooled favorable-outcome proportion was 72.4%, with no statistical heterogeneity detected. However, the small number of contributing cohorts and differences in procedure type, outcome definition, and follow-up preclude interpreting *I*^2^ = 0% as evidence of clinical homogeneity. This estimate was essentially unchanged in the sensitivity analysis restricted to four studies with postoperative seizure outcomes documented at 12 months or longer (72.8%; 95% CI, 65.3%–79.3%).

Clinical response to RFTC was associated with numerically higher odds of favorable outcome after subsequent surgery, but the comparative evidence was limited to three observational cohorts. The conventional random-effects estimate was imprecise and included the null, and Hartung–Knapp small-sample inference further widened the 95% confidence interval to 0.19–77.91. In addition, the magnitude of the association varied substantially in leave-one-out analysis. These findings support only an exploratory association between RFTC response and later surgical outcome and do not establish independent prognostic value.

Approximately one quarter of patients proceeded to a subsequent epilepsy procedure, but this pooled proportion should be interpreted as a descriptive summary rather than a generalizable probability of later intervention. The substantial heterogeneity likely reflects differences in center-specific treatment intent: in some centers, RFTC was offered as definitive or palliative treatment, whereas in others it was used as an intermediate intervention within a broader surgical pathway. RFTC itself was associated with clinically meaningful seizure reduction in approximately three fifths of patients and seizure freedom in nearly half, although both outcomes were heterogeneous. The strict quantitative safety analysis suggested a low pooled proportion of permanent neurological complications, at 2.7%, but this estimate was based on only three studies and sparse events.

Collectively, these findings suggest that SEEG-guided RFTC may occupy several positions within epilepsy care: definitive treatment for selected focal substrates, palliation when larger surgery is considered unsafe, and a functional test of an SEEG-derived surgical hypothesis. Its clinical role should therefore be interpreted according to the underlying substrate, treatment intent, lesion coverage, and broader surgical strategy rather than regarded as uniform across all patients.

### Interpretation and comparison with previous evidence

Previous evidence syntheses have primarily evaluated seizure control and complications after RFTC itself. The earlier meta-analysis by Bourdillon et al. reported variable efficacy across anatomical and etiological subgroups, whereas the more recent analysis by Pichardo-Rojas et al. estimated a 1-year seizure-freedom rate of 37.8% and a responder rate of 69.7% [13, 16]. Pediatric and focal cortical dysplasia–specific reviews have similarly suggested clinically meaningful benefit while emphasizing heterogeneity and the generally limited certainty of the underlying observational evidence [14, 15]. The therapeutic estimates in the present review are broadly compatible with this literature, although direct numerical comparison is limited by differences in follow-up duration, response definitions, etiological composition, and the handling of repeated RFTC and subsequent surgery.

The principal contribution of the present study is its focus on the clinical pathway after RFTC. The favorable outcomes observed after subsequent surgery demonstrate that meaningful seizure control remains achievable in patients who undergo definitive treatment after thermocoagulation. This is particularly relevant because RFTC is frequently offered to patients with complex, deep, MRI-negative, multifocal, or eloquent-cortex epilepsies who may already have a lower probability of straightforward surgical candidacy. Nevertheless, the pooled favorable-outcome proportion should not be interpreted as the expected result for every patient receiving RFTC. Patients who ultimately proceeded to surgery represented a selected subgroup in whom the multidisciplinary team retained sufficient confidence in the localization hypothesis, identified an acceptable treatment target, and anticipated a favorable balance between benefit and procedural risk.

A plausible explanation for the observed responder–outcome association is that thermocoagulation functions as a localized perturbation of the presumed epileptogenic network. Improvement after lesioning selected SEEG contacts may indicate that the treated tissue lies within a network necessary for seizure generation or early propagation. Even a transient response may be informative when the limited lesion volume is insufficient for durable seizure control, because recurrence may reflect incomplete spatial coverage rather than incorrect localization. This interpretation is consistent with reports in which temporary or partial benefit after RFTC contributed to the rationale for subsequent resection or ablation [3, 5, 9, 18–20, 25, 26].

The converse interpretation requires greater caution. Absence of clinical improvement after RFTC does not necessarily invalidate the SEEG-derived hypothesis. A negative response may result from incomplete sampling, restricted lesion volume, avoidance of eloquent or vascular structures, treatment of only one component of a distributed network, or inadequate interruption of early propagation pathways. Several studies reported favorable outcomes after later surgery despite limited or absent benefit from RFTC, and previous technical reviews have similarly emphasized that current evidence does not support using RFTC nonresponse alone to exclude resection when the complete electroclinical evaluation otherwise supports an accessible epileptogenic zone [2, 7, 8, 28, 29]. Preliminary pediatric evidence also suggests that surgery may remain beneficial after unsuccessful thermocoagulation, although abstract-only observations require cautious interpretation [30]. RFTC response may therefore be more useful as a positive confirmatory signal than as a negative exclusionary test.

The prognostic information provided by RFTC may also depend on the magnitude and duration of response rather than on a single binary responder classification. Bourdillon et al. found that early response provided substantial positive predictive information, whereas Chipaux et al. suggested that more durable efficacy was associated with favorable later resection outcomes [9, 25]. Panadés-de Oliveira et al. reported high positive but limited negative predictive value, and both Shields et al. and Du et al. identified the interval to seizure recurrence after RFTC as potentially informative for delayed surgery [3, 18, 19]. In the present meta-analysis, reducing heterogeneous response trajectories to a dichotomous exposure may have obscured clinically relevant information and contributed to the wide confidence interval surrounding the pooled odds ratio.

The leave-one-out findings reinforce this caution. Although the overall point estimate favored responders, the association was substantially attenuated after omission of the largest contributing cohort. The responder-versus-nonresponder association should therefore be considered exploratory and hypothesis-generating rather than evidence of established prognostic value.

### Electrophysiological and network-level interpretation

Clinical response is only one possible marker of whether RFTC has engaged clinically relevant epileptogenic tissue. Changes in interictal spikes, high-frequency oscillations, cross-frequency measures, stimulation-induced discharges, and network connectivity may provide more immediate evidence of target engagement. Contento et al. observed that reductions in electrophysiological biomarkers after thermocoagulation were associated with later resective outcomes even when clinical improvement after RFTC alone was not clearly associated with surgical success [17]. Other cohorts have related RFTC outcome to low-voltage fast activity, stimulation-induced habitual seizures, postcoagulation discharge reduction, or changes in epileptogenic-zone spike rates [1, 31–33].

More recent investigations have extended this framework toward multimodal and network-based prediction. Huang et al. identified anatomical target characteristics associated with RFTC outcome in MRI-negative epilepsy, Kreinter et al. emphasized the importance of disrupting the complete seizure-onset network rather than partially lesioning selected contacts, and Xu et al. reported an association between multimodal diagnostic concordance and post-RFTC seizure outcome [4, 12, 34]. Shen et al. further examined whether post-RFTC changes in SEEG network features could support early machine-learning prediction of treatment outcome [35]. These studies did not provide sufficiently comparable estimates for quantitative synthesis, but they suggest that the eventual prognostic value of RFTC may depend on integrating clinical response with objective evidence of electrophysiological and network disruption.

The anatomical substrate is also likely to modify treatment response. Periventricular nodular heterotopia and other discrete, intrinsically epileptogenic lesions may be particularly amenable to focused thermocoagulation, whereas MRI-negative or broadly distributed networks are more difficult to cover completely [2, 11, 36]. PET positivity and multimodal concordance have been associated with improved localization or RFTC outcomes in MRI-negative and pediatric cohorts, although imaging findings alone have not consistently provided sufficient prognostic discrimination [27, 37, 38]. Target-specific investigations have also suggested that ablation volume and contact coverage within the amygdala or rhinal cortex may influence outcomes in mesial temporal epilepsy, whereas long-term follow-up indicates that early seizure freedom may decline over time [39, 40]. Hypothalamic hamartoma cohorts have identified distinct SEEG patterns associated with outcome, and eloquent motor-cortex series demonstrate that therapeutic benefit may remain possible even when the permissible ablation volume is constrained by function [40, 41].

These observations support a network-informed interpretation of RFTC but caution against treating all epilepsy substrates as biologically equivalent. Historical and technology-adjacent reports illustrate the evolution from relatively focal stereotactic lesioning toward SEEG-defined, multimodal, and increasingly volumetric strategies [6, 7, 29, 31, 42–45]. Future prognostic models will probably require substrate- and target-specific interpretation rather than application of a single response threshold across all patients.

### Clinical implications

The present findings support consideration of SEEG-guided RFTC as a decision-informing intervention in carefully selected patients, but not as an obligatory test before epilepsy surgery. A substantial or sustained response may increase confidence that the treated contacts represent a clinically important component of the epileptogenic network and may help refine the target for resection, disconnection, or laser ablation. This information may be particularly valuable when the hypothesized network is deep, MRI-negative, multifocal, or adjacent to eloquent cortex and when the consequences of an incorrect larger intervention are considerable.

RFTC response should nevertheless be interpreted alongside the complete SEEG recording, electroclinical concordance, functional mapping, structural and functional imaging, extent of lesion coverage, and surgical feasibility. It should not override compelling evidence from the remainder of the presurgical evaluation. This is especially important in temporal lobe epilepsy, in which direct comparison has suggested that RFTC may be less effective than established resective surgery, while subsequent anterior temporal lobectomy may remain effective after RFTC failure [28]. Similarly, absence of a response should not automatically lead to abandonment of an otherwise coherent and surgically actionable hypothesis.

The quantitative safety analysis suggested that permanent neurological complications were uncommon, with a pooled proportion of 2.7%. However, only three studies provided sufficiently consistent data for this analysis, and adverse-event definitions and attribution were heterogeneous across the wider evidence base. The estimate should therefore not be interpreted as a definitive procedure-wide complication rate. Most reported neurological symptoms were transient, but persistent motor, sensory, language, or visual deficits occurred in a minority of patients, particularly when thermocoagulation involved eloquent cortex, visual pathways, or other functionally critical structures [1, 3, 10, 16, 20, 45]. The potential diagnostic or prognostic value of RFTC therefore does not remove the need for detailed vascular review, stimulation mapping, conservative contact selection, and explicit discussion of target-specific risk.

### Future directions

The most important next step is a prospective, multicenter study designed specifically around the prognostic question. Existing cohorts were generally developed to evaluate RFTC efficacy, with subsequent surgery and its outcomes reported as secondary observations. Future studies should prespecify the prognostic exposure, including seizure freedom, percentage seizure reduction, time to first recurrence, response durability, and changes in seizure severity. Standardized assessments at clinically meaningful intervals, such as 1, 3, and 6 months, would permit evaluation of whether early, sustained, or time-dependent response provides the greatest prognostic information.

Later-surgery outcomes should be reported at uniform postoperative timepoints and stratified by procedure type. Resection, disconnection, laser ablation, repeat RFTC, and neuromodulation should not be combined without separate reporting. Studies should also document why patients proceeded, or did not proceed, to further surgery because treatment selection is inseparable from the observed outcome. A prospective decision-impact design could record the intended surgical plan before RFTC, whether RFTC changed confidence in the localization or planned extent of treatment, and whether those changes translated into improved clinical outcomes.

Patient-level technical data will be essential. These should include electrode coordinates, thermolesion volume, the proportion of the seizure-onset zone treated, involvement of early propagation sites, distance from eloquent cortex or vessels, and spatial overlap between thermocoagulated tissue and the final surgical cavity. Integration of these variables with MRI and PET concordance, pathology, clinical response, time to recurrence, and post-RFTC SEEG biomarkers could support multivariable prediction rather than reliance on a single responder label.

Prognostic models should report calibration, discrimination, positive and negative predictive values, and clinical net benefit, followed by external validation in independent centers. An especially informative design would compare models based on routine presurgical information with models that additionally incorporate RFTC response and postlesion electrophysiology. Such studies could determine whether RFTC provides meaningful incremental information beyond SEEG interpretation alone. Shared patient-level datasets and common outcome definitions would also permit individual-participant-data meta-analysis, etiology-specific modeling, and more reliable evaluation of pediatric, MRI-negative, temporal, extratemporal, and eloquent-cortex subgroups.

### Limitations

Several limitations should be considered. First, the evidence was predominantly retrospective and observational, and only six studies contributed to the pooled subsequent-surgery outcome. The responder-versus-nonresponder analysis was restricted to three studies, produced a wide confidence interval, and was sensitive to the inclusion of individual cohorts. The pooled prognostic association therefore remains exploratory.

Second, patients proceeding to later surgery were highly selected. Response to RFTC may itself have influenced the decision to offer surgery, creating confounding by indication and potential incorporation bias. Differences between responders and nonresponders may also reflect lesion visibility, localization confidence, target accessibility, the proportion of the epileptogenic network treated, or the extent of the planned definitive procedure rather than an independent prognostic effect of RFTC response.

Third, studies varied in the definition and timing of RFTC response, favorable postoperative outcome, and follow-up. Engel classes I–II and ILAE classes 1–3 are clinically related but not identical, and use of the longest reported follow-up may not fully account for temporal changes in seizure outcome. Subsequent procedures were also heterogeneous. The sensitivity analysis restricted to studies with postoperative outcomes documented at 12 months or longer yielded an essentially unchanged pooled estimate (72.8%; 95% CI, 65.3%–79.3%), but it included only four studies and does not eliminate heterogeneity in procedure type or favorable-outcome definition. Although no statistical heterogeneity was observed for the favorable later-surgery proportion, the limited number of contributing studies reduces the ability of *I*^2^ and related statistics to identify clinically important differences.

Fourth, most analyses relied on aggregate data. Adjusted prognostic estimates were uncommon, and electrophysiological, imaging, anatomical, and technical predictors could not be incorporated into a unified multivariable model. Overlapping institutional populations were assessed carefully, but incomplete reporting of recruitment periods and patient-level characteristics means that residual overlap cannot be excluded.

Fifth, the pooled safety estimate was derived from only three studies and four permanent events. Incomplete, inconsistent, or selective adverse-event reporting may have led to underestimation of permanent morbidity, and the absence of observed heterogeneity should not be interpreted as evidence of uniform risk across anatomical targets.

Finally, formal assessment of small-study effects was not appropriate for the sparse primary, prognostic, and safety analyses. Item-level JBI appraisal identified limitations typical of retrospective case series and cohort studies, particularly incomplete control of confounding and uncertainty in aspects of participant selection and follow-up. The QUIPS assessment further identified confounding as a high-risk domain in all three studies contributing to the prognostic meta-analysis, reinforcing the exploratory interpretation of the responder–outcome association.

## Conclusion

Among selected patients who underwent subsequent resective, disconnective, or ablative epilepsy surgery after SEEG-guided RFTC, approximately seven in 10 had a favorable seizure outcome. This pooled estimate should be interpreted in the context of differences in subsequent procedure type, favorable-outcome definition, and follow-up across the contributing studies. Clinical response to RFTC was associated with numerically higher odds of favorable later surgical outcome, but the comparative evidence was sparse, imprecise, and sensitive to individual studies. RFTC response may therefore provide useful confirmatory information within a broader multimodal surgical evaluation, particularly when localization is complex; however, current evidence does not support using RFTC response or nonresponse as a standalone criterion for offering or withholding definitive epilepsy surgery. Prospective multicenter studies incorporating response magnitude and duration, post-RFTC electrophysiology, lesion coverage, and standardized later-surgery outcomes are required before independent prognostic utility can be established for clinical decision-making.

## Supplementary Information

Below is the link to the electronic supplementary material.

Supplementary file1 Figure S1. Sensitivity analysis restricted to studies with postoperative outcomes assessed at 12 months or longer. Random-effects meta-analysis of favorable seizure outcome after qualifying subsequent epilepsy surgery, restricted to four studies in which postoperative seizure outcomes were documented at 12 months or longer for the contributing surgical cohort. Favorable outcomes occurred in 113 of 155 patients. The pooled favorable-outcome proportion was 72.8% (95% confidence interval, 65.3%–79.3%), with no statistical heterogeneity detected (*I*² = 0%; *τ*² <.0001; *Q* = 0.61, df = 3; *P* =.893). Squares represent study-specific proportions, horizontal lines indicate 95% confidence intervals, and the diamond represents the pooled estimate. CI, confidence interval; RFTC, radiofrequency thermocoagulation; SEEG, stereoelectroencephalography. (PDF 33 KB)

Supplementary file2 Figure S2. Leave-one-out analysis of the association between RFTC response and favorable subsequent-surgery outcome. Influence analysis showing the pooled random-effects odds ratio after sequential omission of each of the three studies contributing to the principal prognostic model. The pooled odds ratio was 8.88 (95% confidence interval, 2.62–30.14) after omission of Chipaux et al., 4.27 (95% confidence interval, 0.57–31.96) after omission of Panadés-de Oliveira et al., and 1.49 (95% confidence interval, 0.32–7.00) after omission of Bourdillon et al. The variation across iterations demonstrates the sensitivity of the prognostic estimate to individual studies. CI, confidence interval; OR, odds ratio; RFTC, radiofrequency thermocoagulation. (PDF 5 KB)

Supplementary file3 Figure S3. Proportion of patients proceeding to a subsequent epilepsy procedure after SEEG-guided RFTC. Random-effects meta-analysis of 23 studies reporting progression to a subsequent epilepsy procedure after RFTC. The pooled proportion was 22.0% (95% confidence interval, 11.1%–38.8%), with substantial between-study heterogeneity (*I*² = 84.1%). The analysis included heterogeneous clinical pathways in which RFTC was used as definitive treatment, palliation, or an intermediate step before an additional intervention. Squares represent study-specific proportions, horizontal lines indicate 95% confidence intervals, and the diamond represents the pooled estimate. CI, confidence interval; RFTC, radiofrequency thermocoagulation; SEEG, stereoelectroencephalography. (PDF 28 KB)

Supplementary file4 Figure S4. Clinically meaningful seizure response after SEEG-guided RFTC. Random-effects meta-analysis of 17 studies reporting clinically meaningful seizure reduction after RFTC. Clinical response was most commonly defined as a reduction of at least 50% in seizure frequency, although study-specific definitions and assessment timepoints varied. The pooled responder proportion was 61.3% (95% confidence interval, 53.0%–69.0%), with substantial heterogeneity (*I*² = 75.2%). Squares represent study-specific proportions, horizontal lines indicate 95% confidence intervals, and the diamond represents the pooled estimate. CI, confidence interval; RFTC, radiofrequency thermocoagulation; SEEG, stereoelectroencephalography. (PDF 33 KB)

Supplementary file5 Figure S5. Seizure freedom after SEEG-guided RFTC. Random-effects meta-analysis of 17 studies reporting seizure freedom after RFTC. The pooled seizure-freedom proportion was 46.8% (95% confidence interval, 36.0%–58.0%), with considerable between-study heterogeneity (*I*² = 82.7%). Follow-up duration and the definition and timing of seizure-freedom assessment varied among studies. Squares represent study-specific proportions, horizontal lines indicate 95% confidence intervals, and the diamond represents the pooled estimate. CI, confidence interval; RFTC, radiofrequency thermocoagulation; SEEG, stereoelectroencephalography. (PDF 31 KB)

Supplementary file6 Figure S6. Permanent neurological complications attributable to SEEG-guided RFTC. Random-intercept logistic generalized linear mixed model of permanent neurological complications reported in three independent studies comprising 150 patients. Four permanent complications were recorded. The pooled permanent-complication proportion was 2.7% (95% confidence interval, 1.0%–6.9%), with no observed statistical heterogeneity (*I*² = 0%; *τ*² = 0; Wald *χ*² = 0.93; *P* =.628). The small number of studies and sparse event count limit the precision of the pooled estimate and heterogeneity assessment. Squares represent study-specific proportions, horizontal lines indicate 95% confidence intervals, and the diamond represents the pooled estimate. CI, confidence interval; GLMM, generalized linear mixed model; RFTC, radiofrequency thermocoagulation; SEEG, stereoelectroencephalography. (PDF 5 KB)

Supplementary file7 Figure S7. Funnel plot of clinically meaningful seizure response after SEEG-guided RFTC. Funnel plot of the 17 studies contributing to the meta-analysis of clinically meaningful seizure response after RFTC. Study estimates are displayed on the logit scale against their standard errors. The vertical dashed line represents the pooled effect estimate, and the diagonal lines indicate the approximate pseudo-95% confidence limits. The plot was interpreted descriptively because substantial clinical heterogeneity, variation in response definitions and assessment timepoints, and proportions near the boundaries may contribute to asymmetry independently of publication bias. RFTC, radiofrequency thermocoagulation; SEEG, stereoelectroencephalography. (PDF 27 KB)

Supplementary file8 Figure S8. Funnel plot of seizure freedom after SEEG-guided RFTC. Funnel plot of the 17 studies contributing to the meta-analysis of seizure freedom after RFTC. Study estimates are displayed on the logit scale against their standard errors. The vertical dashed line represents the pooled effect estimate, and the diagonal lines indicate the approximate pseudo-95% confidence limits. Visual asymmetry was interpreted cautiously because follow-up duration, seizure-freedom definitions, clinical populations, and boundary-event frequencies varied considerably among studies. RFTC, radiofrequency thermocoagulation; SEEG, stereoelectroencephalography. (PDF 26 KB)

Supplementary file9 Figure S9. Funnel plot of progression to a subsequent epilepsy procedure after SEEG-guided RFTC. Funnel plot of the 23 studies contributing to the meta-analysis of progression to a subsequent epilepsy procedure after RFTC. Study estimates are displayed on the logit scale against their standard errors. The vertical dashed line represents the pooled effect estimate, and the diagonal lines indicate the approximate pseudo-95% confidence limits. Visual asymmetry was interpreted descriptively because the intended role of RFTC, follow-up duration, access to subsequent surgery, and definitions of eligible downstream interventions differed substantially among studies. RFTC, radiofrequency thermocoagulation; SEEG, stereoelectroencephalography. (PDF 27 KB)

Supplementary file10 Table S1. Prespecified eligibility criteria for the systematic review and meta-analysis. Eligibility criteria are presented according to the population, intervention, comparator, outcomes, and study-design framework. The table specifies the criteria applied to original clinical studies, the handling of contextual and citation-searching sources, subsequent procedures, favorable seizure outcomes, RFTC response, and quantitative eligibility. RFTC, radiofrequency thermocoagulation; SEEG, stereoelectroencephalography. Table S2. Reports excluded after full-text assessment and reasons for exclusion. The table lists the 93 reports excluded after full-text assessment and the primary prespecified reason for each exclusion. Exclusion categories comprised absence of an extractable outcome meeting the prespecified therapeutic, prognostic, safety, or downstream-treatment eligibility criteria (*n* = 23); ancillary, technical, or biomarker outcomes only (*n* = 15); review, survey, or background publication (*n* = 22); SEEG or epilepsy surgery without RFTC as the index intervention (*n* = 8); case report or very small case series (*n* = 7); ineligible intervention or outcome (*n* = 10); conference abstract or short report (*n* = 6); overlapping cohort (*n* = 1); and non-peer-reviewed preprint (*n* = 1). RFTC, radiofrequency thermocoagulation; SEEG, stereoelectroencephalography. Table S3. Characteristics of studies contributing to the primary later-surgery meta-analysis. The table summarizes the subsequent procedure, number of patients, favorable-outcome definition, favorable-outcome numerator and denominator, and postoperative follow-up for the six studies contributing to the primary favorable later-surgery meta-analysis. Chandra et al. is additionally noted because its downstream-intervention cohort included both qualifying resective/disconnective procedures and vagus nerve stimulation, while favorable outcomes were not reported separately for the qualifying procedures; it was therefore excluded from the strict primary meta-analysis. RFTC, radiofrequency thermocoagulation; VNS, vagus nerve stimulation. (DOCX 35 KB)

Supplementary file11 PRISMA 2020 checklist. Completed PRISMA 2020 checklist indicating the location within the manuscript or supplementary materials where each reporting item is addressed. (DOCX 273 KB)

Supplementary file12 Complete database search strategies. Full reproducible search strategies used for PubMed/MEDLINE, Embase, Scopus, and Web of Science, including all search terms, Boolean operators, field restrictions, filters, and the date of the final search. (DOCX 13 KB)

Supplementary file13 Study-level extraction and analysis dataset. Complete study-level data extraction file containing study characteristics, patient and intervention variables, analytical-role classifications, outcome-specific eligibility assignments, analysis-ready datasets, and reconstructed contingency tables used for the prognostic and quantitative syntheses. (XLSX 145 KB)

Supplementary file14 Risk-of-bias assessments of included studies and prognostic evidence. Complete item-level Joanna Briggs Institute critical appraisal assessments for the 29 included original clinical studies and domain-level Quality In Prognosis Studies assessments, with supporting rationales, for the three studies contributing to the responder-versus-nonresponder prognostic meta-analysis. JBI judgments were not converted to numerical scores or overall risk-of-bias categories. (XLSX 12 KB)

Supplementary file15 Title and abstract screening decisions. Complete record-level title and abstract screening dataset, including the eligibility decision and exclusion reason assigned to each record excluded during the initial screening stage. (XLSX 166 KB)

## Data Availability

All data generated or analyzed during this study are included in this article and its supplementary information files. The complete study-level extraction dataset, analytical-role classifications, analysis-ready datasets, reconstructed contingency tables, risk-of-bias assessments, and title and abstract screening decisions are provided in Supplementary Files 3–5. No individual patient-level data were generated or collected.

## Declarations

### PROSPERO registration

CRD420261430081

### Author approval statement

All authors contributed to the work, reviewed and approved the final version of the manuscript, and agree with its submission to *Epilepsia*.

## References

[1] Catenoix H et al (2015) Seizures outcome after stereoelectroencephalography-guided thermocoagulations in malformations of cortical development poorly accessible to surgical resection. Neurosurgery 77(1):9–14 10.1227/NEU.0000000000000723 26086907

[2] Mirandola L et al (2017) Stereo-EEG: diagnostic and therapeutic tool for periventricular nodular heterotopia epilepsies. Epilepsia 58(11):1962–1971 10.1111/epi.13895 28880999

[3] Panadés-De Oliveira L et al (2023) Stereo-electroencephalography–guided radiofrequency thermocoagulation in patients with MRI-negative focal epilepsy. J Neurosurg 138(3):837–846 10.3171/2022.6.JNS22733 35962969

[4] Huang Q et al (2024) Predictors of seizure outcomes in stereo-electroencephalography-guided radio-frequency thermocoagulation for MRI-negative epilepsy. Ther Adv Chronic Dis 15. 10.1177/20406223241236258 PMC10943718 38496233

[5] Cossu M et al (2015) Stereoelectroencephalography-guided radiofrequency thermocoagulation in the epileptogenic zone: a retrospective study on 89 cases. J Neurosurg 123(6):1358–1367 10.3171/2014.12.JNS141968 26090841

[6] Cossu M et al (2017) Stereo-EEG–guided radiofrequency thermocoagulations. Epilepsia 58:66–72 10.1111/epi.13687 28386919

[7] Bourdillon P et al (2020) Surgical techniques: stereoelectroencephalography-guided radiofrequency-thermocoagulation (SEEG-guided RF-TC). Seizure 77:64–68 10.1016/j.seizure.2019.01.021 30711397

[8] Dimova P et al (2017) Radiofrequency thermocoagulation of the seizure-onset zone during stereoelectroencephalography. Epilepsia 58(3):381–392 10.1111/epi.13663 28150296

[9] Bourdillon P et al (2017) Stereo electroencephalography–guided radiofrequency thermocoagulation (SEEG-guided RF-TC) in drug-resistant focal epilepsy: results from a 10-year experience. Epilepsia 58(1):85–93 10.1111/epi.13616 27859033

[10] Xu Y et al (2022) Stereoelectroencephalography-guided radiofrequency thermocoagulation of epileptic foci in the eloquent motor cortex: feasibility, safety, and efficacy. World Neurosurg 164:e492–e500 10.1016/j.wneu.2022.04.133 35537694

[11] Slegers R et al (2024) Stereo-electroencephalography-guided radiofrequency thermocoagulation restricted to periventricular nodular heterotopias in patients with drug-resistant epilepsy: a single center experience. Seizure 121:105–113 10.1016/j.seizure.2024.07.016 39146706

[12] Kreinter H et al (2024) Disrupting the epileptogenic network with stereoelectroencephalography-guided radiofrequency thermocoagulation. Epilepsia 65(7):e113–e118 10.1111/epi.18005 38738924

[13] Bourdillon P et al (2018) Stereo-electroencephalography-guided radiofrequency thermocoagulation in patients with focal epilepsy: a systematic review and meta-analysis. Epilepsia 59(12):2296–2304 10.1111/epi.14584 30345535

[14] Li Y et al (2024) Magnetic resonance-guided laser interstitial thermal therapy vs. stereoelectroencephalography-guided radiofrequency thermocoagulation in epilepsy patients with focal cortical dysplasia: a systematic review and meta-analysis. Front Neurol 14. 10.3389/fneur.2023.1241763 PMC10625445 37928136

[15] Bottan JS et al (2025) Radiofrequency-thermocoagulation in pediatric epilepsy surgery: a systematic review and pooled analysis of cases. Seizure 126:6–13 10.1016/j.seizure.2025.01.012 39874864

[16] Pichardo-Rojas PS et al (2025) Stereoelectroencephalography-guided radiofrequency thermocoagulation for drug-resistant epilepsy: a meta-analysis. Epilepsia 66(10):3602–3620 10.1111/epi.18524 40569267

[17] Contento M et al (2021) Changes in epileptogenicity biomarkers after stereotactic thermocoagulation. Epilepsia 62(9):2048–2059 10.1111/epi.16989 34272883

[18] Shields JA et al (2023) Stereoelectroencephalography-guided radiofrequency ablation of the epileptogenic zone as a treatment and predictor of future success of further surgical intervention. Epilepsia 64(8):2081–2093 10.1111/epi.17673 PMC11051685 37300533

[19] Du C et al (2024) Stereoelectroencephalography-guided radiofrequency thermocoagulation of the epileptogenic zone: a potential treatment and prognostic indicator for subsequent excision surgery. Acta Neurochir 166(1). 10.1007/s00701-024-06106-x 38735896

[20] Chandra PS et al (2025) SEEG-guided radio-frequency thermocoagulation of the epileptogenic networks: its utility for both treatment and validation for localizing epileptogenic networks. Stereotact Funct Neurosurg 103(5):312–325 10.1159/000548196 40889253

[21] Page MJ et al (2021) The PRISMA 2020 statement: an updated guideline for reporting systematic reviews. BMJ 372:n71 10.1136/bmj.n71 PMC8005924 33782057

[22] Viechtbauer W (2010) Conducting meta-analyses in R with the metafor package. J Stat Softw 36(3):1–48

[23] Balduzzi S, Rücker G, Schwarzer G (2019) How to perform a meta-analysis with R: a practical tutorial. Evid Based Ment Health 22(4):153–160 10.1136/ebmental-2019-300117 PMC10231495 31563865

[24] Wickham H (2016) Data analysis. In: Wickham H (ed) Ggplot2: elegant graphics for data analysis. Springer International Publishing, Cham, pp 189–201

[25] Chipaux M et al (2019) SEEG-guided radiofrequency thermocoagulation of epileptic foci in the paediatric population: feasibility, safety and efficacy. Seizure 70:63–70 10.1016/j.seizure.2019.07.004 31288205

[26] Vasquez A et al (2025) Radiofrequency thermocoagulation in focal epilepsy: a retrospective cohort study. Epilepsia Open 10(2):529–538 10.1002/epi4.70009 PMC12014932 40018762

[27] Chen W et al (2026) Clinical outcomes of seeg-guided radiofrequency thermocoagulation in children with focal drug-resistant epilepsy: a multicenter real-world study. Ann Clin Transl Neurol. 10.1002/acn3.70373 PMC13395011 41888055

[28] Moles A et al (2018) SEEG-guided radiofrequency coagulation (SEEG-guided RF-TC) versus anterior temporal lobectomy (ATL) in temporal lobe epilepsy. J Neurol 265(9):1998–2004 10.1007/s00415-018-8958-9 29943202

[29] Catenoix H et al (2018) The combination of stereo-EEG and radiofrequency ablation. Epilepsy Res 142:117–120 10.1016/j.eplepsyres.2018.01.012 29336937

[30] Tugbiyele MA et al (2023) Stereoelectroencephalograhy-guided radiofrequency thermocoagulation in paediatric patients with refractory epilepsy: a single-center prospective cohort. Brain Spine 3:102271

[31] Zhao Q et al (2017) Stereotactic bilateral transfrontal minimal radiofrequency thermocoagulation of the amygdalohippocampal complex for bilateral medial temporal lobe epilepsy: a retrospective study of 12 patients. Epileptic Disord 19(2):152–165 10.1684/epd.2017.0914 28637635

[32] Liu C et al (2021) Stereoelectroencephalography-guided radiofrequency thermocoagulation for hypothalamic hamartoma: electroclinical patterns and the relationship with surgical prognosis. Epilepsy Behav 118:107957 10.1016/j.yebeh.2021.107957 33872942

[33] Yan J et al (2025) Quantitative research of epileptogenicity biomarkers and early prognosis after stereoscopic electroencephalography guided radiofrequency thermocoagulation in drug-resistant epilepsy patients. Medicine (Baltimore) 104(30):e43334 10.1097/MD.0000000000043334 PMC12303451 40725932

[34] Xu Z et al (2026) Multimodal diagnostic concordance and seizure outcomes following stereo-encephalography-guided volumetric radiofrequency thermocoagulation in drug-resistant epilepsy. Epilepsy Res 219. 10.1016/j.eplepsyres.2025.107691 41218353

[35] Shen J et al (2026) The SEEG brain network predicts epileptic surgical outcomes of radiofrequency thermocoagulation. Epilepsy Res 219. 10.1016/j.eplepsyres.2025.107715 41330179

[36] Liu Q et al (2024) SEEG-RFTC in patients with refractory focal epilepsy: real-world outcomes from 121 cases. Ann Clin Transl Neurol 11(8):1999–2007 10.1002/acn3.52117 PMC11330216 38968332

[37] Li H et al (2023) Utility of hybrid PET/MRI in stereoelectroencephalography guided radiofrequency thermocoagulation in MRI negative epilepsy patients. Front Neurosci 17. 10.3389/fnins.2023.1163946 PMC10291085 37378015

[38] Chen W et al (2025) Stereo-electroencephalography-guided radiofrequency thermocoagulation in children with MRI-negative epilepsy. Epilepsy Behav 172. 10.1016/j.yebeh.2025.110741 41045786

[39] Jean S et al (2024) Optimizing outcomes in drug-resistant mesial temporal lobe epilepsy patients undergoing stereoelectroencephalography-guided radiofrequency thermocoagulation. Ther Adv Neurol Disord 17. 10.1177/17562864241286867 PMC11472416 39403115

[40] Li K et al (2024) Stereo-electroencephalography-guided three-dimensional radiofrequency thermocoagulation for mesial temporal lobe epilepsy with hippocampal sclerosis: a retrospective study with long-term follow-up. Epilepsia Open 9(3):918–925 10.1002/epi4.12866 PMC11145609 37968869

[41] Liu C et al (2025) Stereo-electroencephalography pattern and long-term seizure outcome in hypothalamic hamartoma treated by radiofrequency thermocoagulation. J Neurosurg 142(6):1558–1565 10.3171/2024.9.JNS241261 39919283

[42] Wu Z et al (2014) Efficacy and safety of a new robot-assisted stereotactic system for radiofrequency thermocoagulation in patients with temporal lobe epilepsy. Exp Ther Med 7(6):1728–1732 10.3892/etm.2014.1620 PMC4043615 24926375

[43] Méreaux JL et al (2020) Practice of stereoelectroencephalography (sEEG) in drug-resistant epilepsy: retrospective series with surgery and thermocoagulation outcomes. Neurochirurgie 66(3):139–143 10.1016/j.neuchi.2019.12.014 32278000

[44] Castellano JF et al (2024) Stereoelectroencephalography-guided radiofrequency thermocoagulation: diagnostic and therapeutic implications. Brain Sci 14(2). 10.3390/brainsci14020110 PMC10887298 38391685

[45] Iwasaki M et al (2025) Volume-based stereotactic radiofrequency thermocoagulation for drug-resistant focal epilepsy: preliminary multicenter report from Japan. Oper Neurosurg. Publish Ahead of Print. 10.1227/ons.0000000000001869 PMC13456546 41400514

