# Supplementary material for: Subsequent epilepsy surgery after SEEG-guided radiofrequency thermocoagulation: Outcomes and prognostic value of treatment response—a systematic review and meta-analysis
Source: Neurosurg Rev. 2026 Nov 6;49(1):638. doi: 10.1007/s10143-026-04561-x (PMC13740120; doi:10.1007/s10143-026-04561-x)
Supplement: Supplementary file 1 [file 10143_2026_4561_MOESM1_ESM.pdf]

Sensitivity analysis restricted to ≥12-month postoperative follow-up

Favorable later-surgery outcome: 72.8% [95% CI 65.3–79.3]; $I^2 = 0.0\%$

Study	Favorable / total	Proportion [95% CI]	Weight
**Overall random-effects**	**113/155**	**72.8% [65.3, 79.3]**	**100.0%**
Bourdillon 2017	67/91	73.6% [63.3, 82.3]	57.9%
Moles 2018	14/19	73.7% [48.8, 90.9]	12.1%
Contento 2021	16/21	76.2% [52.8, 91.8]	12.5%
Du 2024	16/24	66.7% [44.7, 84.4]	17.5%

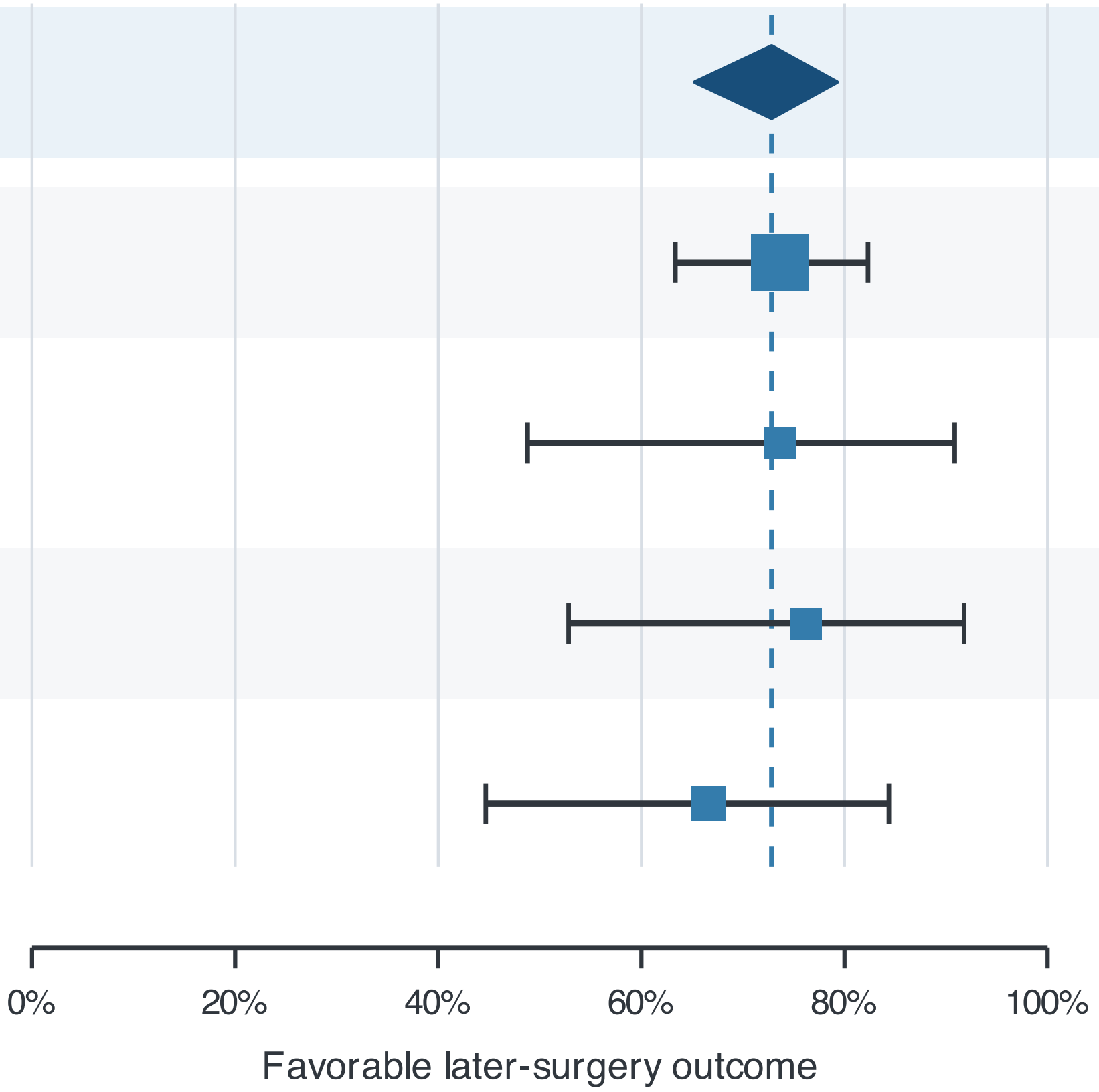

Sensitivity analysis included only studies in which postoperative seizure outcomes were documented at ≥12 months.

Heterogeneity: $I^2 = 0.0\%$; $\tau^2 < 0.0001$; $Q = 0.61$, $df = 3$, $P = 0.893$
