# Supplementary material for: Subsequent epilepsy surgery after SEEG-guided radiofrequency thermocoagulation: Outcomes and prognostic value of treatment response—a systematic review and meta-analysis
Source: Neurosurg Rev. 2026 Nov 6;49(1):638. doi: 10.1007/s10143-026-04561-x (PMC13740120; doi:10.1007/s10143-026-04561-x)
Supplement: Supplementary file 2 [file 10143_2026_4561_MOESM2_ESM.pdf]

Leave−one−out analysis of the prognostic association between RFTC response and later−surgery outcome

Overall random−effects model: OR = 3.86 [95% CI 0.80–18.57]; I^2 = 47.1%

Analysis	OR [95% CI]	P value	$.^2$	.	I^2
**Overall random−effects model**	**3.86 [0.80, 18.57]**	0.0924	0.9358	0.9674	47.1%
Omitting Chipaux 2019	8.88 [2.62, 30.14]	0.0005	0	0	0%
Omitting Panadés−de Oliveira 2023	4.27 [0.57, 31.96]	0.1576	1.5133	1.2302	71.3%
Omitting Bourdillon 2017	1.49 [0.32, 7.00]	0.6105	0	0	0%

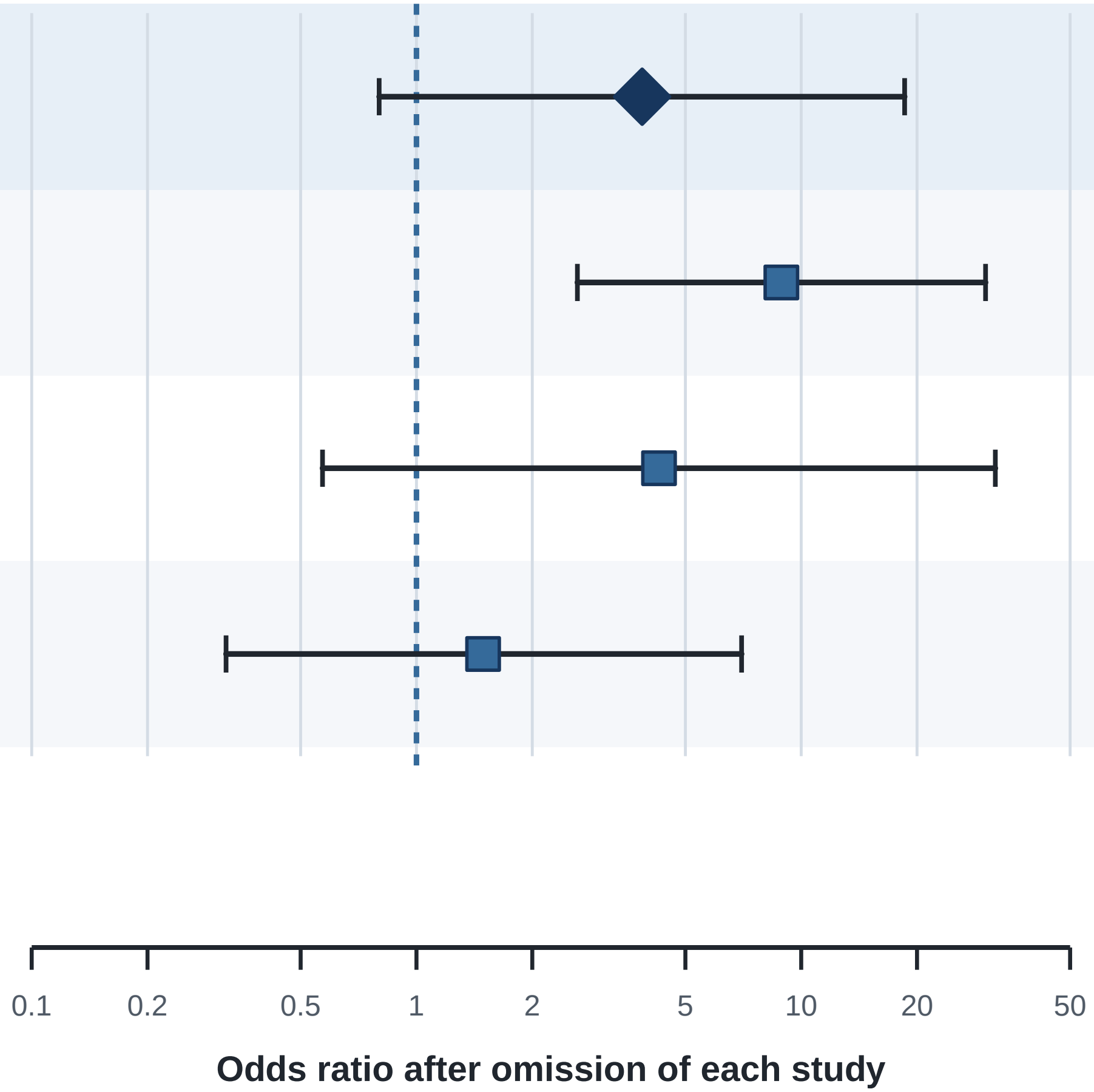

Values >1 favor a better later−surgery outcome among RFTC responders.
