# Supplementary material for: Subsequent epilepsy surgery after SEEG-guided radiofrequency thermocoagulation: Outcomes and prognostic value of treatment response—a systematic review and meta-analysis
Source: Neurosurg Rev. 2026 Nov 6;49(1):638. doi: 10.1007/s10143-026-04561-x (PMC13740120; doi:10.1007/s10143-026-04561-x)
Supplement: Supplementary file 3 [file 10143_2026_4561_MOESM3_ESM.pdf]

Subsequent epilepsy procedure after SEEG-guided RFTC

Random-effects model: 22.0% [95% CI 11.1–38.8]; I² = 84.1%

Study	Events/total	Estimate [95% CI]
Xu 2022	1/15	6.7% [0.9, 35.2]
Xu 2026	0/63	0.0% [0.0, 11.3]
Shen 2026	10/31	32.3% [18.3, 50.3]
Chipaux 2019	30/46	65.2% [50.5, 77.5]
Slegers 2024	0/24	0.0% [0.1, 25.1]
Liu 2021	0/27	0.0% [0.1, 23.0]
Chen 2025	0/33	0.0% [0.1, 19.6]
Jean 2024	0/22	0.0% [0.1, 26.8]
Cossu 2015	51/89	57.3% [46.9, 67.1]
Liu 2024	39/121	32.2% [24.5, 41.0]
Chen 2026	0/111	0.0% [0.0, 6.7]
Li 2024	2/35	5.7% [1.4, 20.2]
Vasquez 2025	20/25	80.0% [60.0, 91.4]
Bourdillon 2017	91/162	56.2% [48.4, 63.6]
Dimova 2017	9/23	39.1% [21.8, 59.8]
Contento 2021	21/38	55.3% [39.5, 70.1]
Kreinter 2024	6/18	33.3% [15.8, 57.1]
Li 2023	0/27	0.0% [0.1, 23.0]
Huang 2024	12/30	40.0% [24.3, 58.1]
Yan 2025	16/44	36.4% [23.6, 51.4]
Catenoix 2015	0/14	0.0% [0.2, 36.6]
Moles 2018	19/21	90.5% [68.9, 97.6]
Chandra 2025	29/61	47.5% [35.4, 60.0]
**Pooled estimate**		**22.0% [11.1, 38.8]**

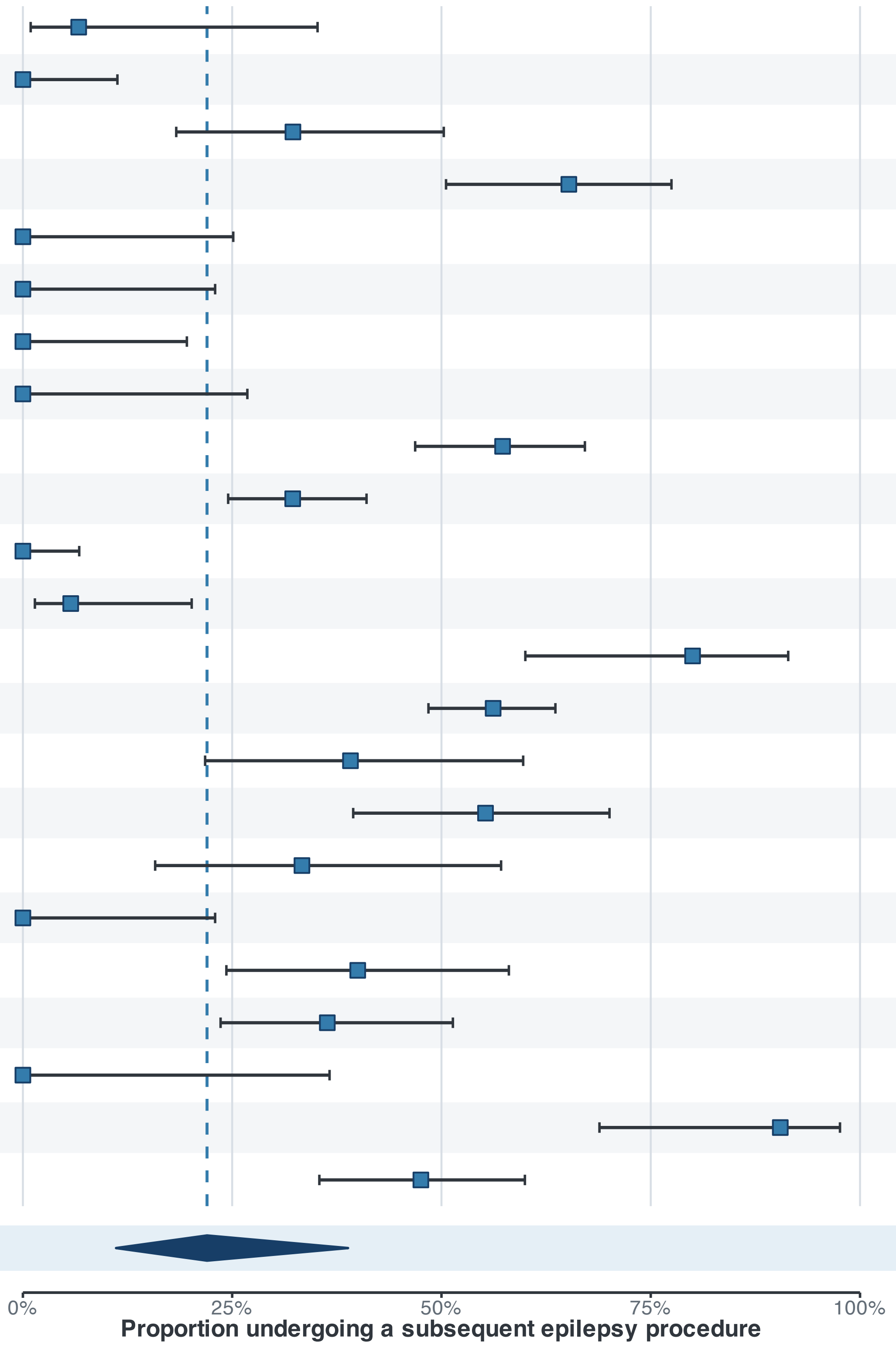

The pooled proportion is interpreted descriptively because subsequent treatment pathways varied substantially across studies.
