# Supplementary material for: Subsequent epilepsy surgery after SEEG-guided radiofrequency thermocoagulation: Outcomes and prognostic value of treatment response—a systematic review and meta-analysis
Source: Neurosurg Rev. 2026 Nov 6;49(1):638. doi: 10.1007/s10143-026-04561-x (PMC13740120; doi:10.1007/s10143-026-04561-x)
Supplement: Supplementary file 4 [file 10143_2026_4561_MOESM4_ESM.pdf]

Therapeutic response after SEEG-guided RFTC

Random-effects model: 61.3% [95% CI 53.0–69.0]; I^2 = 75.2%

Study	Events/total	Estimate [95% CI]
Méreaux 2020	7/8	87.5% [46.3, 98.3]
Xu 2022	12/15	80.0% [53.0, 93.4]
Xu 2026	41/63	65.1% [52.6, 75.8]
Shen 2026	17/31	54.8% [37.4, 71.1]
Slegers 2024	17/24	70.8% [50.2, 85.4]
Liu 2021	23/27	85.2% [66.5, 94.3]
Chen 2025	25/33	75.8% [58.5, 87.4]
Jean 2024	16/22	72.7% [51.1, 87.2]
Cossu 2015	25/89	28.1% [19.7, 38.3]
Liu 2024	82/121	67.8% [59.0, 75.5]
Chen 2026	73/111	65.8% [56.5, 74.0]
Li 2024	22/35	62.9% [46.0, 77.1]
Vasquez 2025	12/25	48.0% [29.6, 66.9]
Dimova 2017	8/23	34.8% [18.4, 55.7]
Contento 2021	19/38	50.0% [34.6, 65.4]
Catenoix 2015	9/14	64.3% [37.6, 84.3]
Moles 2018	10/21	47.6% [27.9, 68.2]
**Pooled estimate**		**61.3% [53.0, 69.0]**

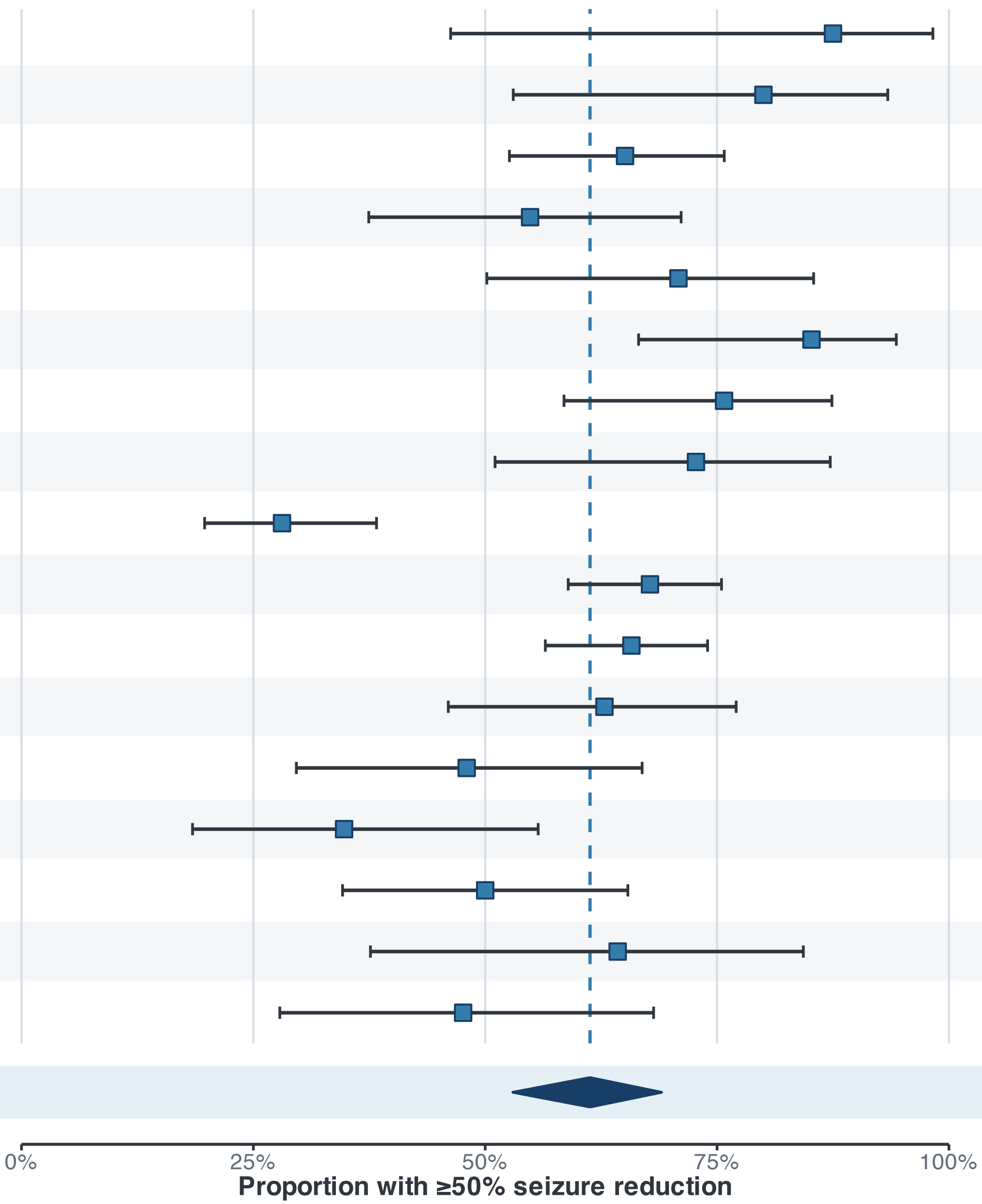

Clinically meaningful response was defined most commonly as a reduction of ≥50% in seizure frequency.
