# Supplementary material for: Subsequent epilepsy surgery after SEEG-guided radiofrequency thermocoagulation: Outcomes and prognostic value of treatment response—a systematic review and meta-analysis
Source: Neurosurg Rev. 2026 Nov 6;49(1):638. doi: 10.1007/s10143-026-04561-x (PMC13740120; doi:10.1007/s10143-026-04561-x)
Supplement: Supplementary file 5 [file 10143_2026_4561_MOESM5_ESM.pdf]

## Seizure freedom after SEEG-guided RFTC

Random-effects model: 46.8% [95% CI 36.0–58.0]; I² = 82.7%

| Study | Events/total | Estimate [95% CI] |
|---|---|---|
| Méreaux 2020 | 3/8 | 37.5% [12.5, 71.5] |
| Xu 2022 | 9/15 | 60.0% [34.8, 80.8] |
| Xu 2026 | 41/63 | 65.1% [52.6, 75.8] |
| Shen 2026 | 17/31 | 54.8% [37.4, 71.1] |
| Slegers 2024 | 11/24 | 45.8% [27.5, 65.4] |
| Liu 2021 | 19/27 | 70.4% [51.0, 84.4] |
| Chen 2025 | 17/33 | 51.5% [34.9, 67.8] |
| Jean 2024 | 16/22 | 72.7% [51.1, 87.2] |
| Cossu 2015 | 16/89 | 18.0% [11.3, 27.4] |
| Liu 2024 | 54/121 | 44.6% [36.0, 53.6] |
| Chen 2026 | 73/111 | 65.8% [56.5, 74.0] |
| Vasquez 2025 | 3/25 | 12.0% [3.9, 31.3] |
| Contento 2021 | 8/38 | 21.1% [10.9, 36.8] |
| Li 2023 | 15/27 | 55.6% [36.9, 72.8] |
| Huang 2024 | 11/30 | 36.7% [21.6, 54.9] |
| Moles 2018 | 0/21 | 0.0% [0.1, 27.7] |
| Mirandola 2017 | 13/17 | 76.5% [51.4, 90.9] |
| **Pooled estimate** | | **46.8% [36.0, 58.0]** |

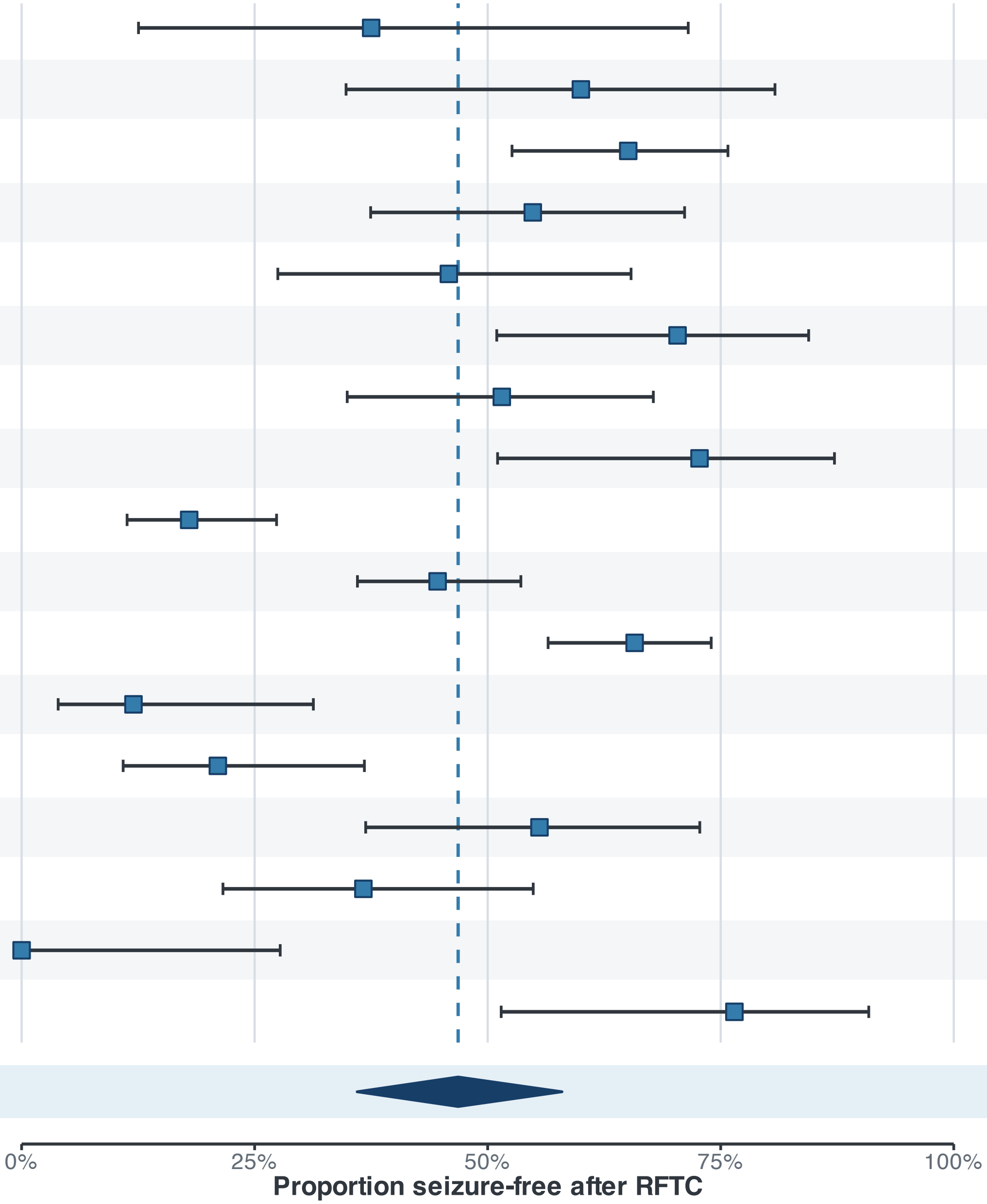


Seizure freedom was defined according to each study's reported RFTC outcome definition and assessment timepoint.
