# Supplementary material for: Subsequent epilepsy surgery after SEEG-guided radiofrequency thermocoagulation: Outcomes and prognostic value of treatment response—a systematic review and meta-analysis
Source: Neurosurg Rev. 2026 Nov 6;49(1):638. doi: 10.1007/s10143-026-04561-x (PMC13740120; doi:10.1007/s10143-026-04561-x)
Supplement: Supplementary file 6 [file 10143_2026_4561_MOESM6_ESM.pdf]

Permanent neurological complications after SEEG−guided RFTC

Random−effects model: 2.7% [95% CI 1.0−6.9]; $I^2 = 0.0\%$

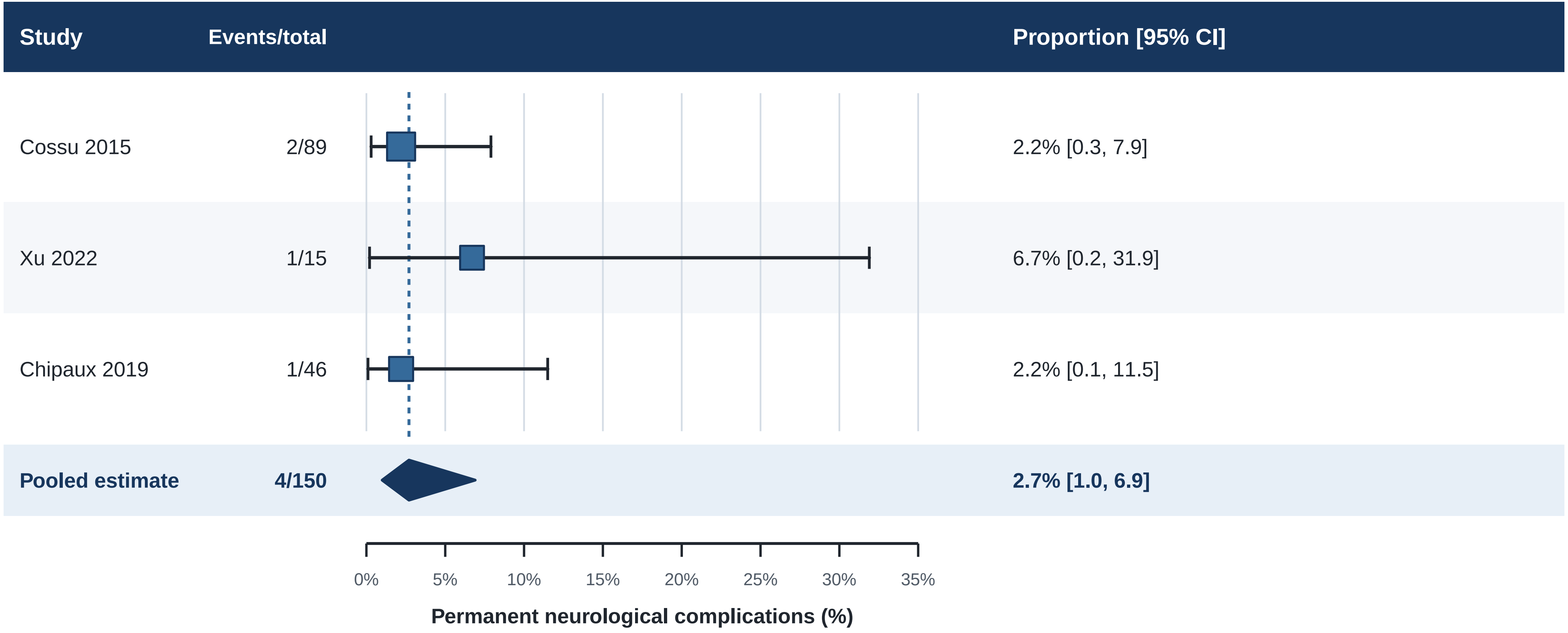

Heterogeneity: $I^2 = 0.0\%$; .^{2} = 0; Q = 0.93, df = 2, P = 0.628
