# Supplementary material for: Subsequent epilepsy surgery after SEEG-guided radiofrequency thermocoagulation: Outcomes and prognostic value of treatment response—a systematic review and meta-analysis
Source: Neurosurg Rev. 2026 Nov 6;49(1):638. doi: 10.1007/s10143-026-04561-x (PMC13740120; doi:10.1007/s10143-026-04561-x)
Supplement: Supplementary file 7 [file 10143_2026_4561_MOESM7_ESM.pdf]

Funnel plot for clinically meaningful seizure response after SEEG-guided RFTC

Random-effects pooled response: 61.3% [95% CI 53.0–69.0]; I² = 75.2%

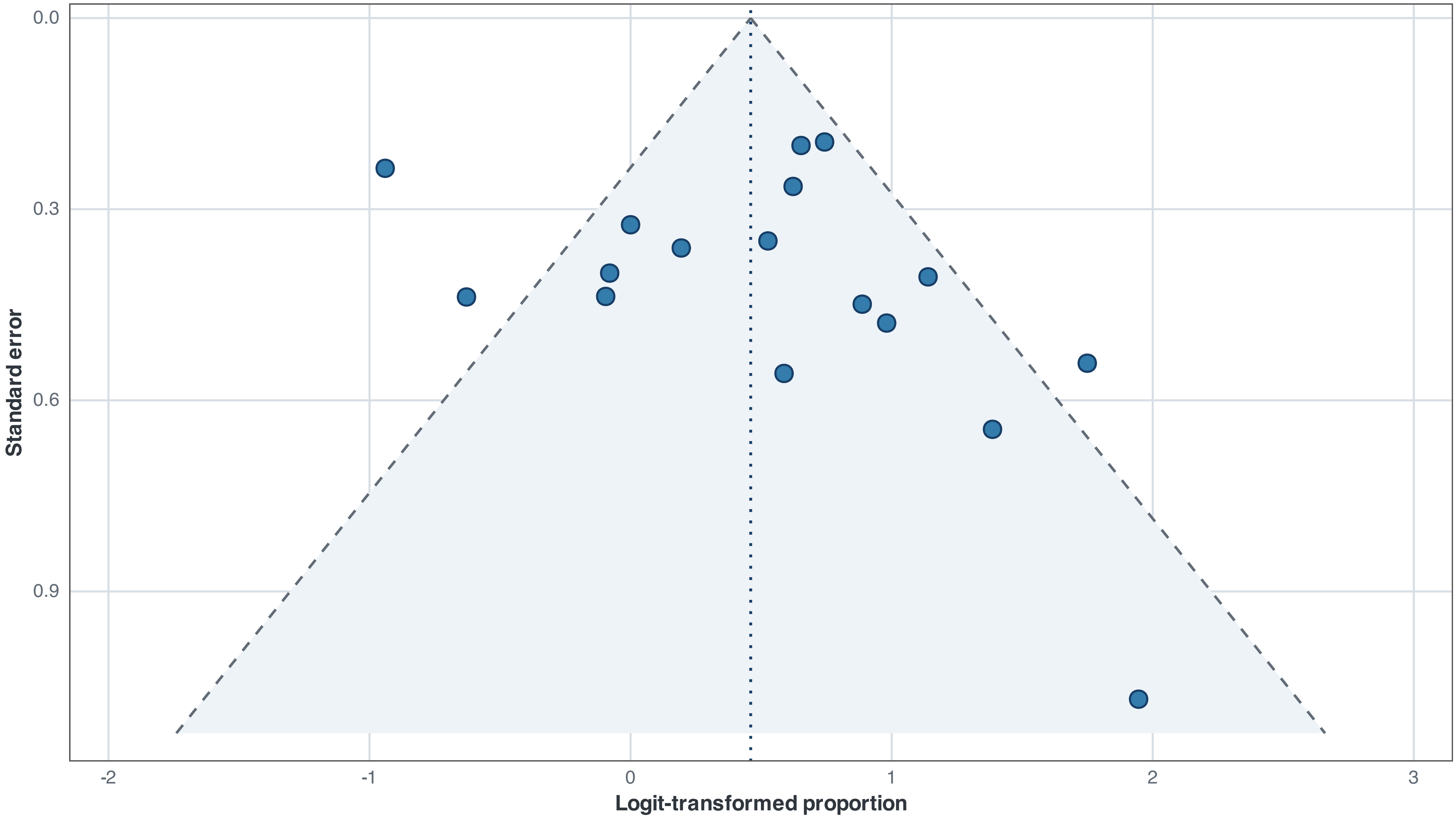

The dotted vertical line represents the pooled logit; the dashed boundaries indicate the approximate 95% funnel region.
