# Supplementary material for: Subsequent epilepsy surgery after SEEG-guided radiofrequency thermocoagulation: Outcomes and prognostic value of treatment response—a systematic review and meta-analysis
Source: Neurosurg Rev. 2026 Nov 6;49(1):638. doi: 10.1007/s10143-026-04561-x (PMC13740120; doi:10.1007/s10143-026-04561-x)
Supplement: Supplementary file 8 [file 10143_2026_4561_MOESM8_ESM.pdf]

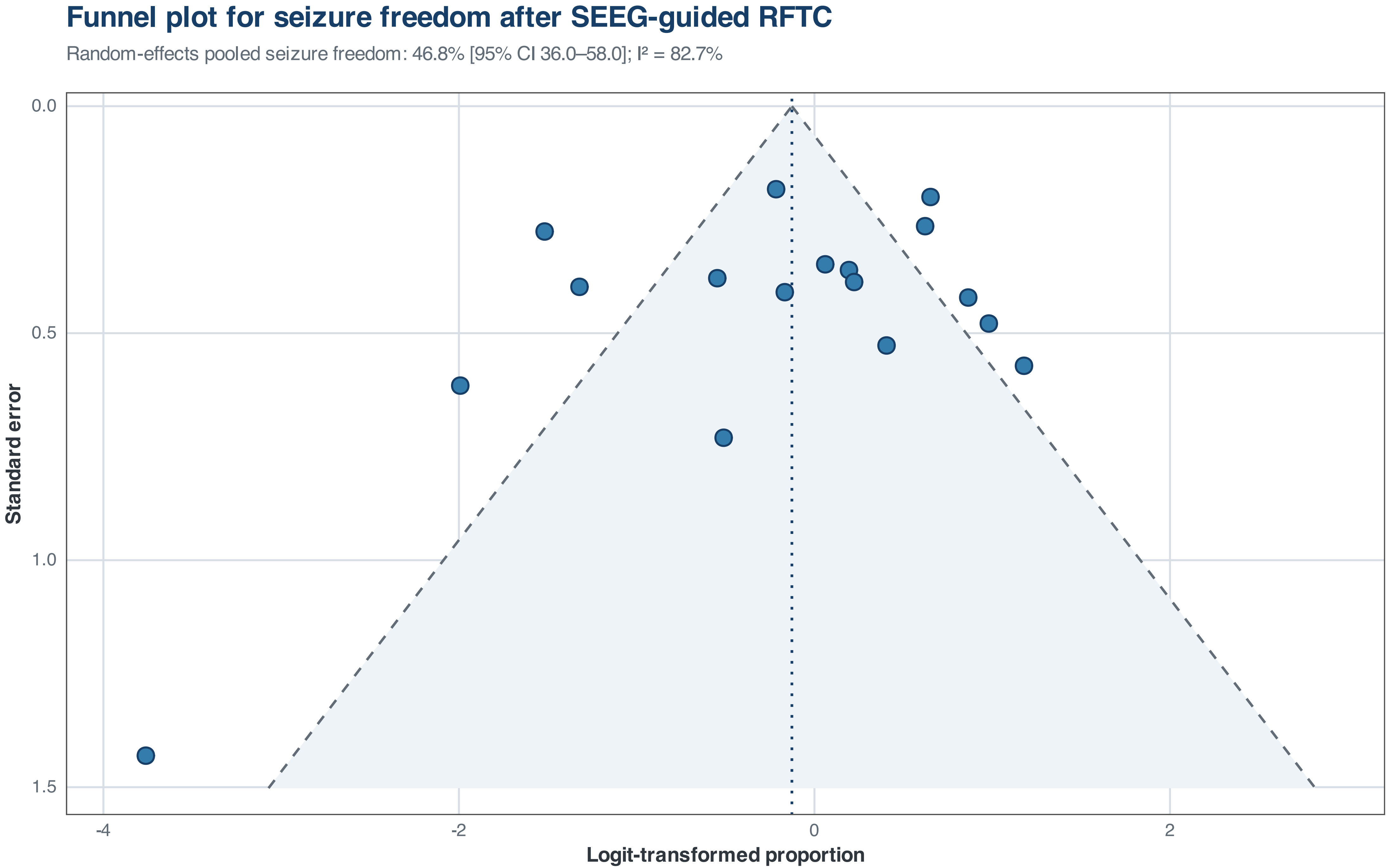

The dotted vertical line represents the pooled logit; the dashed boundaries indicate the approximate 95% funnel region.
