# Supplementary material for: Subsequent epilepsy surgery after SEEG-guided radiofrequency thermocoagulation: Outcomes and prognostic value of treatment response—a systematic review and meta-analysis
Source: Neurosurg Rev. 2026 Nov 6;49(1):638. doi: 10.1007/s10143-026-04561-x (PMC13740120; doi:10.1007/s10143-026-04561-x)
Supplement: Supplementary file 9 [file 10143_2026_4561_MOESM9_ESM.pdf]

Funnel plot for subsequent epilepsy procedure after SEEG-guided RFTC

Random-effects pooled proportion: 22.0% [95% CI 11.1–38.8]; I² = 84.1%

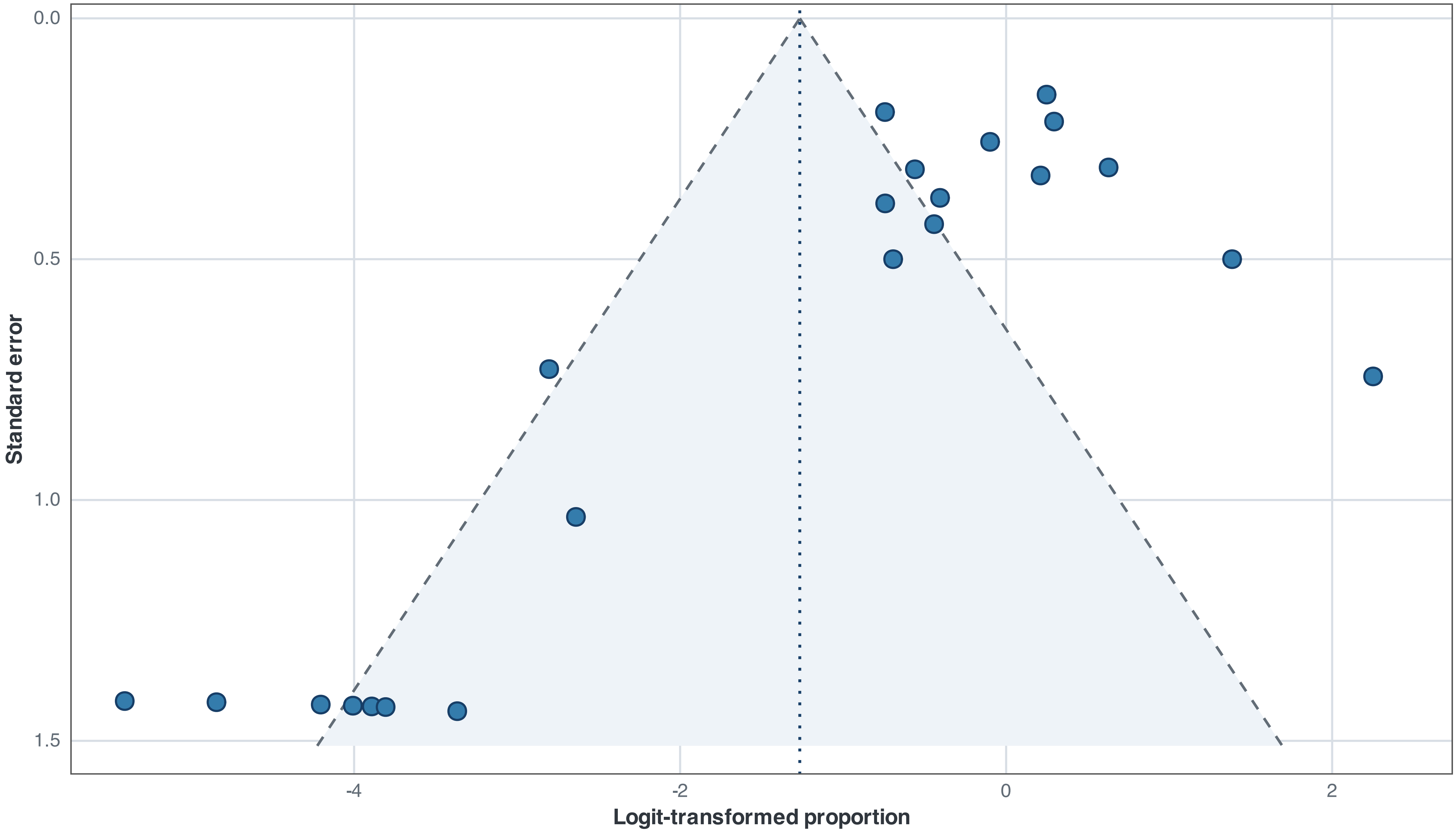

The dotted vertical line represents the pooled logit; dashed lines indicate the approximate 95% funnel region. Interpretation is descriptive because of substantial heterogeneity.
