# Supplementary material for: Subsequent epilepsy surgery after SEEG-guided radiofrequency thermocoagulation: Outcomes and prognostic value of treatment response—a systematic review and meta-analysis
Source: Neurosurg Rev. 2026 Nov 6;49(1):638. doi: 10.1007/s10143-026-04561-x (PMC13740120; doi:10.1007/s10143-026-04561-x)
Supplement: Supplementary file 10 [file 10143_2026_4561_MOESM10_ESM.docx]

**Supplementary Table S1. Prespecified eligibility criteria for the systematic review and meta-analysis**

| **Domain** | **Inclusion criteria** | **Exclusion criteria** |
| --- | --- | --- |
| **Population** | Children, adults, or mixed-age populations with drug-resistant focal epilepsy undergoing SEEG-guided RFTC. Eligible populations included temporal, extratemporal, lesional, MRI-negative, multifocal, and network-based epilepsies. | Populations without drug-resistant focal epilepsy; studies in which data for the eligible SEEG-RFTC population could not be extracted separately. |
| **Index intervention** | Radiofrequency thermocoagulation or radiofrequency ablation performed through stereotactically implanted intracerebral electrodes used for SEEG evaluation. Alternative terminology was accepted when the procedural description confirmed treatment through SEEG electrode contacts. | RFTC not performed through SEEG electrodes; open radiofrequency lesioning, conventional stereotactic lesioning, laser ablation, resection, neuromodulation, or another intervention used as the index treatment. |
| **Subsequent intervention** | Lesionectomy, tailored cortical resection, temporal resection, selective amygdalohippocampectomy, disconnective surgery, laser interstitial thermal therapy, or another definitive stereotactic ablative procedure performed after SEEG-guided RFTC. | Procedures performed before or independently of RFTC. Neuromodulatory procedures were documented as part of the treatment pathway but were not included in the strict primary later-surgery outcome. |
| **Comparator** | No comparator required for single-arm analyses. For the prognostic analysis, RFTC responders were compared with RFTC nonresponders. | Studies without extractable responder-versus-nonresponder data were excluded only from the comparative prognostic meta-analysis, not necessarily from the overall review. |
| **RFTC response definition** | Preferentially defined as a reduction of ≥50% in seizure frequency or temporary or sustained seizure freedom. Clearly reported study-specific definitions were accepted. | No clinically interpretable or extractable definition of post-RFTC response. |
| **Primary outcome** | Favorable seizure outcome after subsequent resective, disconnective, or ablative epilepsy surgery following SEEG-guided RFTC, preferentially Engel classes I–II or ILAE classes 1–3. Comparable author-defined favorable outcomes were accepted when standard classifications were unavailable. | No extractable postoperative seizure outcome after an eligible subsequent procedure. |
| **Principal prognostic outcome** | Association between clinical response to RFTC and favorable seizure outcome after subsequent surgery. | No extractable comparative effect estimate or reconstructable \(2 \times 2\) contingency table. |
| **Secondary outcomes** | Progression to a subsequent epilepsy procedure; clinically meaningful seizure reduction after RFTC; seizure freedom after RFTC; duration of RFTC benefit; and permanent neurological complications attributable to SEEG-guided RFTC. | No extractable eligible therapeutic, prognostic, safety, or downstream-treatment outcome. |
| **Follow-up** | Longest available postoperative follow-up used when several assessment timepoints were reported. | Follow-up insufficient to determine the relevant clinical outcome. |
| **Eligible study designs** | Prospective or retrospective cohort studies, comparative observational studies, and case series reporting extractable cohort-level clinical outcomes. | Case reports; very small case series without analytically usable cohort-level data; animal or laboratory studies; protocols; editorials; correspondence without original clinical data; and purely technical studies without eligible patient outcomes. |
| **Contextual evidence** | Relevant systematic reviews, meta-analyses, narrative reviews, technical syntheses, and abstract-only reports could be used for contextual interpretation and citation searching but were not considered included studies. | These publications were not eligible as original clinical studies and were not counted in the included evidence base. They contributed no independent patient observations or effect estimates to quantitative synthesis. |
|  |  |  |
| **Mixed populations or interventions** | Eligible when data relating specifically to SEEG-guided RFTC could be extracted separately or reliably attributed to the eligible cohort. | Mixed populations or interventions without separately extractable SEEG-RFTC data. |
| **Overlapping cohorts** | The most complete report was selected for each relevant outcome. Different reports could contribute to different analyses when they provided nonoverlapping outcome information. | Duplicate patient observations were not counted more than once within the same meta-analysis. |
| **Language and publication date** | No language or publication-date restrictions. Potentially eligible non-English reports were translated for assessment and extraction. | None based solely on language or publication year. |
| **Quantitative synthesis** | Conducted when at least two clinically compatible original studies reported an extractable numerator and denominator or comparable effect estimate. | Studies without compatible numerical data were retained for narrative synthesis when otherwise eligible. |

**Eligibility criteria are presented according to the population, intervention, comparator, outcomes, and study-design framework. The table specifies the criteria applied to original clinical studies, the handling of contextual and citation-searching sources, subsequent procedures, favorable seizure outcomes, RFTC response, and quantitative eligibility. RFTC, radiofrequency thermocoagulation; SEEG, stereoelectroencephalography.**

**SUPPLEMENTARY TABLE S2. Reports excluded after full-text assessment and reasons for exclusion.**

| Selection_Role | First_Author | Year | Title | Journal | DOI | Exclusion_Reason |
| --- | --- | --- | --- | --- | --- | --- |
| Conference abstract | Balatskaya | 2022 | SEEG-guided radiofrequency thermocoagulation in refractory focal epilepsy | European Journal of Neurology | 10.1111/ene.15466 | Conference abstract or short meeting report; insufficient full-report methodological and outcome information for inclusion as an original clinical study. |
| Review/background | Wang | 2020 | Optimized stereoelectroencephalography-guided radiofrequency thermocoagulation in the treatment of patients with focal epilepsy | Annals of Translational Medicine | 10.21037/atm.2019.10.112 | Review/technical background publication; not an original extractable clinical cohort for the planned synthesis. |
| Case report/small case | Zhengbo | 2026 | Stereoelectroencephalography-guided radiofrequency thermocoagulation for secondary occipital lobe epilepsy in a child: a case report | Child's Nervous System | 10.1007/s00381-025-07114-7 | Case report or very small case-based report; insufficient cohort-level data for quantitative synthesis. |
| Review/background | Negida | 2022 | The Efficacy of Stereoelectroencephalography-Guided Radiofrequency Thermocoagulation in Patients with Drug-Resistant Epilepsy; A Meta-Analysis of 331 Patients' Data | Stereotactic and Functional Neurosurgery | 10.1159/000526735 | Review, survey, or background article; not an original extractable cohort for the planned synthesis. |
| Overlapping cohort | Wang | 2022 | Stereoelectroencephalography-guided radiofrequency thermocoagulation in drug-resistant focal epilepsy | Annals of Translational Medicine | 10.21037/atm-21-6851 | Earlier Huanhu Hospital SEEG-RFTC cohort with substantial overlap with the later, larger Du 2024 cohort from the same center and author group; excluded to avoid double-counting, with the more complete later report retained for the relevant outcomes. |
| Conference abstract | Wang | 2024 | SEEG guided thermocoagulation for refractory epilepsy - Indications and outcome | British Journal of Neurosurgery | 10.1080/02688697.2023.2269001 | Conference proceeding abstract; insufficient full-report methodological and outcome information for inclusion as an original clinical study. |
| Near-miss SEEG-RFTC outcome | Deng | 2020 | Efficacy of stereoelectroencephalography-guided radiofrequency thermocoagulation in the treatment of drug-resistant epilepsy in children: A report of 71 cases | Chinese Journal of Neurosurgery | 10.3760/cma.j.cn112050-20191012-00438 | SEEG/RFTC therapeutic, efficacy, safety, or subtype-specific study; no extractable later resective/ablative surgery outcome for the target analysis. |
| Near-miss SEEG-RFTC outcome | Minkin | 2024 | Monopolar stereoelectroencephalography-guided radiofrequency thermocoagulation | Journal of Neurosurgery | 10.3171/2023.7.JNS23319 | SEEG/RFTC therapeutic, efficacy, safety, or subtype-specific study; no extractable later resective/ablative surgery outcome for the target analysis. |
| Review/background | Ramantani | 2026 | Pediatric epilepsy surgery: Global survey of invasive explorations | Epilepsia | 10.1002/epi.70325 | Review, survey, or background article; not an original extractable cohort for the planned synthesis. |
| Case report/small case | Wei | 2022 | Case Report: Stereoelectroencephalography and Stereoelectroencephalography-Guided Radiofrequency Thermocoagulation in Familial Lateral Temporal Lobe Epilepsy | Frontiers in Neurology | 10.3389/fneur.2022.864070 | Case report or very small case-based report; insufficient cohort-level data for quantitative synthesis. |
| Other related epilepsy surgery | Liu | 2020 | Electroclinical features of lateral and medial orbitofrontal epilepsy: a case series | Epileptic Disorders | 10.1684/epd.2020.1230 | Related epilepsy surgery or presurgical evaluation article, but wrong intervention/outcome for this review. |
| Other related epilepsy surgery | Pelliccia | 2024 | Tailored surgical work-up in patients with periventricular nodular heterotopias | Epilepsia | 10.1111/epi.18151 | Related epilepsy surgery or presurgical evaluation article, but wrong intervention/outcome for this review. |
| SEEG/surgery without RFTC | Cho | 2025 | Stereoelectroencephalographic exploration and surgical outcome in Lennox–Gastaut syndrome | Epilepsia | 10.1111/epi.18283 | SEEG diagnostic or epilepsy surgery outcome study without SEEG-guided RFTC as the index intervention. |
| Near-miss SEEG-RFTC outcome | Wei | 2018 | Stereoelectroencephalography-Guided Radiofrequency Thermocoagulation for Hypothalamic Hamartomas: Preliminary Evidence | World Neurosurgery | 10.1016/j.wneu.2018.03.148 | SEEG/RFTC therapeutic, efficacy, safety, or subtype-specific study; no extractable later resective/ablative surgery outcome for the target analysis. |
| Near-miss SEEG-RFTC outcome | Xu | 2018 | The application of stereo-electroencephalography guided radiofrequency thermocoagulation in refractory temporal epilepsy | National Medical Journal of China | 10.3760/cma.j.issn.0376-2491.2018.13.011 | SEEG/RFTC therapeutic, efficacy, safety, or subtype-specific study; no extractable later resective/ablative surgery outcome for the target analysis. |
| Near-miss SEEG-RFTC outcome | Liu | 2022 | Utility of Hybrid PET/MRI in Stereoelectroencephalography-Guided Radiofrequency Thermocoagulation in Patients with Drug-Resistant Epilepsy | Stereotactic and Functional Neurosurgery | 10.1159/000526735 | SEEG/RFTC therapeutic, efficacy, safety, or subtype-specific study; no extractable later resective/ablative surgery outcome for the target analysis. |
| SEEG/surgery without RFTC | Chiarello | 2024 | Negative MRI and a seizure onset zone close to eloquent areas in FCD type II: Application of MRg-LiTT after a SEEG re-evaluation in pediatric patients with a previous failed surgery | Epilepsy and Behavior | 10.1016/j.yebeh.2024.109694 | SEEG diagnostic or epilepsy surgery outcome study without SEEG-guided RFTC as the index intervention. |
| Ancillary/biomarker or technical | Bregianni | 2024 | Psychiatric complications following SEEG-guided radiofrequency thermocoagulations in patients with drug-resistant epilepsy | Epilepsy and Behavior | 10.1016/j.yebeh.2024.109806 | Ancillary biomarker, network, neuropsychological, safety, or technical study; no eligible later-surgery outcome data. |
| Near-miss SEEG-RFTC outcome | Gong | 2025 | A three-dimensional conformal radiofrequency thermocoagulation method for epileptogenic zones in functional cerebral areas: evaluation and prediction of surgical outcomes | Acta Epileptologica | 10.1186/s42494-025-00214-6 | SEEG/RFTC therapeutic, efficacy, safety, or subtype-specific study; no extractable later resective/ablative surgery outcome for the target analysis. |
| Ancillary/biomarker or technical | Shen | 2026 | Alteration of functional connectivity and network properties after stereo-electroencephalography guided radiofrequency thermocoagulation | Chinese Neurosurgical Journal | 10.1186/s41016-026-00428-8 | Ancillary biomarker, network, neuropsychological, safety, or technical study; no eligible later-surgery outcome data. |
| Near-miss SEEG-RFTC outcome | Luo | 2022 | Optimization and high-density array of stereoelectroencephalography-guided radiofrequency thermocoagulation for the treatment of pediatric tuberous sclerosis complex with drug-resistant epilepsy | CNS Neuroscience and Therapeutics | 10.1111/cns.13804 | SEEG/RFTC therapeutic, efficacy, safety, or subtype-specific study; no extractable later resective/ablative surgery outcome for the target analysis. |
| Near-miss SEEG-RFTC outcome | Yan | 2023 | Efficacy and its influencing factors of stereoelectroencephalography-guided radiofrequency thermocoagulation for the treatment of drug-refractory epilepsy | Chinese Journal of Neurosurgery | 10.3760/cma.j.cn112050-20220527-00277 | SEEG/RFTC therapeutic, efficacy, safety, or subtype-specific study; no extractable later resective/ablative surgery outcome for the target analysis. |
| Near-miss SEEG-RFTC outcome | Xia | 2024 | Observation on the long-term efficacy of stereoelectroencephalography-guided radiofrequency thermocoagulation in the treatment of mesial temporal lobe epilepsy | Chinese Journal of Neurosurgery | 10.3760/cma.j.cn112050-20240229-00077 | SEEG/RFTC therapeutic, efficacy, safety, or subtype-specific study; no extractable later resective/ablative surgery outcome for the target analysis. |
| Ancillary/biomarker or technical | Song | 2024 | Diffusion spectrum imaging based semi-automatic optic radiation tractography for vision preservation in SEEG-guided radiofrequency thermocoagulation | Seizure | 10.1016/j.seizure.2023.11.014 | Ancillary biomarker, network, neuropsychological, safety, or technical study; no eligible later-surgery outcome data. |
| Near-miss SEEG-RFTC outcome | Scholly | 2018 | Seeg as unique procedure versus SEEG-guided radiofrequency thermocoagulation in drug-resistant focal epilepsy: Efficacy and safety with regard to long-term outcome | Epilepsia | 10.1111/epi.14612 | SEEG/RFTC therapeutic, efficacy, safety, or subtype-specific study; no extractable later resective/ablative surgery outcome for the target analysis. |
| Other related epilepsy surgery | Wang | 2020 | Magnetic resonance–guided laser interstitial thermal therapy versus stereoelectroencephalography-guided radiofrequency thermocoagulation for drug-resistant focal cortical dysplasia | Epilepsy Research | 10.1016/j.eplepsyres.2020.106397 | Related epilepsy surgery or presurgical evaluation article, but wrong intervention/outcome for this review. |
| Review/background | Yamamoto | 2020 | Recent advancement of technologies and the transition to new concepts in epilepsy surgery | Neurologia Medico-Chirurgica | 10.2176/nmc.ra.2020-0197 | Review, survey, or background article; not an original extractable cohort for the planned synthesis. |
| Case report/small case | Li | 2022 | Case Report: Late-Onset Lennox-Gastaut Syndrome Treated With Stereotactic Electroencephalography-Guided Radiofrequency Thermocoagulation Before Craniofacial Surgery | Frontiers in Neurology | 10.3389/fneur.2022.857767 | Case report or very small case-based report; insufficient cohort-level data for quantitative synthesis. |
| Conference abstract | Moreno | 2026 | PO 191 Safety and efficacy of stereoeeg-guided radiofrequency thermocoagulation for drug-resistant focal epilepsy: a single-center 4-year experience | Clinical Neurophysiology | 10.1016/j.clinph.2025.2111203 | Conference abstract or short meeting report; insufficient extractable full-text data. |
| Ancillary/biomarker or technical | Simula | 2022 | Brain networks functional connectivity changes after stereotactic thermocoagulation in drug-resistant epilepsy | Epilepsia | 10.1111/epi.17388 | Ancillary biomarker, network, neuropsychological, safety, or technical study; no eligible later-surgery outcome data. |
| Ancillary/biomarker or technical | Yang | 2023 | Stereo-Electroencephalography Guided Radiofrequency Thermocoagulation Prognosis Prediction Based on Brain Network Features of Patients with Refractory Epilepsy | Chinese Journal of Biomedical Engineering | 10.3969/j.issn.0258-8021.2023.06.002 | Ancillary biomarker, network, neuropsychological, safety, or technical study; no eligible later-surgery outcome data. |
| Ancillary/biomarker or technical | Simula | 2023 | Changes in local and network brain activity after stereotactic thermocoagulation in patients with drug-resistant epilepsy | Epilepsia | 10.1111/epi.17613 | Ancillary biomarker, network, neuropsychological, safety, or technical study; no eligible later-surgery outcome data. |
| Conference abstract | Van Hoof | 2023 | N°17 – Brain network connectivity in epilepsy patients is affected after stereo-electroencephalography-guided radiofrequency thermocoagulation | Clinical Neurophysiology | 10.1016/j.clinph.2023.03.032 | Conference abstract or short meeting report; insufficient extractable full-text data. |
| Near-miss SEEG-RFTC outcome | Dai | 2025 | Optimized Stereo-Electroencephalography-Guided Three-Dimensional Radiofrequency Thermocoagulation for Hypothalamic Hamartomas-Related Epilepsy: A Single-Center Retrospective Study | CNS Neuroscience and Therapeutics | 10.1111/cns.70462 | SEEG/RFTC therapeutic, efficacy, safety, or subtype-specific study; no extractable later resective/ablative surgery outcome for the target analysis. |
| Preprint/non-peer-reviewed | Iwasaki | 2025 | Lesion-guided stereotactic radiofrequency thermocoagulation for drug-resistant focal epilepsy: preliminary multi-center report from Japan | medRxiv | 10.1101/2025.05.20.25327981 | Preprint/non-peer-reviewed record; not eligible for the primary peer-reviewed synthesis. |
| Review/background | Barba | 2026 | Beyond Broca and Wernicke: Epilepsy surgery in the language areas | Epileptic Disorders | 10.1002/epd2.70282 | Review, survey, or background article; not an original extractable cohort for the planned synthesis. |
| Case report/small case | Liu | 2019 | Multiple Stereoelectroencephalography-Guided Radiofrequency Thermocoagulations for Polymicrogyria With Startle Seizures: A Case Report | Frontiers in Neurology | 10.3389/fneur.2019.01095 | Case report or very small case-based report; insufficient cohort-level data for quantitative synthesis. |
| Review/background | Brigo | 2021 | Camillo Negro (1861–1927) and Antonio Carle (1854–1927): Pioneers of non-resective surgical approach to epilepsy treatment | Epilepsy and Behavior | 10.1016/j.yebeh.2021.108360 | Review, survey, or background article; not an original extractable cohort for the planned synthesis. |
| Case report/small case | De Ribaupierre | 2023 | EEG monitoring during Radiofrequency-thermocoagulation in pediatric epilepsy surgery. Case series and review of the literature | Child's Nervous System | 10.1007/s00381-023-06118-5 | Case report or very small case-based report; insufficient cohort-level data for quantitative synthesis. |
| Ancillary/biomarker or technical | Li | 2023 | Frameless robot-assisted stereoelectroencephalography-guided radiofrequency: methodology, results, complications and stereotactic application accuracy | Frontiers in Neurology | 10.3389/fneur.2023.1259171 | Ancillary biomarker, network, neuropsychological, safety, or technical study; no eligible later-surgery outcome data. |
| Ancillary/biomarker or technical | Gula | 2024 | Connectivity changes after stereo-electroencephalography-guided radiofrequency thermocoagulation are unaffected by the presence of interictal epileptiform discharges | Epilepsia | 10.1111/epi.18151 | Ancillary biomarker, network, neuropsychological, safety, or technical study; no eligible later-surgery outcome data. |
| Near-miss SEEG-RFTC outcome | Fan | 2019 | Optimized SEEG-guided radiofrequency thermocoagulation for mesial temporal lobe epilepsy with hippocampal sclerosis | Seizure | 10.1016/j.seizure.2019.08.011 | SEEG/RFTC therapeutic, efficacy, safety, or subtype-specific study; no extractable later resective/ablative surgery outcome for the target analysis. |
| Review/background | Youngerman | 2019 | Stereoelectroencephalography in epilepsy, cognitive neurophysiology, and psychiatric disease: Safety, efficacy, and place in therapy | Neuropsychiatric Disease and Treatment | 10.2147/NDT.S177804 | Review, survey, or background article; not an original extractable cohort for the planned synthesis. |
| Case report/small case | Ferri | 2023 | A case of clinical worsening after stereo-electroencephalographic-guided radiofrequency thermocoagulation in a patient with polymicrogyria | Epilepsy and Behavior Reports | 10.1016/j.ebr.2022.100579 | Case report or very small case-based report; insufficient cohort-level data for quantitative synthesis. |
| Review/background | Ekman | 2024 | Insular Epilepsy: Functions, Diagnostic Approaches, and Surgical Interventions | Journal of Integrative Neuroscience | 10.31083/j.jin2311209 | Review, survey, or background article; not an original extractable cohort for the planned synthesis. |
| Ancillary/biomarker or technical | Cunha | 2024 | Stereoelectroencephalography monitoring with Epileptogenicity Index and Radiofrequency Thermocoagulation - An intermediary for epilepsy surgery? | Epilepsia | 10.1111/epi.18151 | Ancillary biomarker, network, neuropsychological, safety, or technical study; no eligible later-surgery outcome data. |
| SEEG/surgery without RFTC | Candela-Cantó | 2024 | ROBOTIC STEREOTACTIC SURGERY IN PEDIATRIC PATIENTS: 8 YEARS SINGLE-CENTER EXPERIENCE | Stereotactic and Functional Neurosurgery | NR | SEEG diagnostic or epilepsy surgery outcome study without SEEG-guided RFTC as the index intervention. |
| Review/background | McGonigal | 2025 | Stereoelectroencephalography for Epilepsy Presurgical Assessment: A Nationwide Survey of Evolution of Practice in Australia | Neurology: Clinical Practice | 10.1212/CPJ.0000000000200512 | Review, survey, or background article; not an original extractable cohort for the planned synthesis. |
| Ancillary/biomarker or technical | Catalán-Aguilar | 2026 | Beyond seizure control: The impact of SEEG-guided radiofrequency thermocoagulation on quality of life | Epileptic Disorders | 10.1002/epd2.70201 | Ancillary biomarker, network, neuropsychological, safety, or technical study; no eligible later-surgery outcome data. |
| Review/background | Campbell | 2026 | Stereo-electroencephalography–guided cross-electrode radiofrequency thermocoagulation in focal epilepsy: A review of current methods, technical parameters, and outcome reporting | Epilepsia | 10.1002/epi.70261 | Review, survey, or background article; not an original extractable cohort for the planned synthesis. |
| Ancillary/biomarker or technical | Staudt | 2018 | Radiofrequency energy and electrode proximity influences stereoelectroencephalography-guided radiofrequency thermocoagulation lesion size: An in vitro study with clinical correlation | Operative Neurosurgery | 10.1093/ons/opx291 | Ancillary biomarker, network, neuropsychological, safety, or technical study; no eligible later-surgery outcome data. |
| Near-miss SEEG-RFTC outcome | Gracia | 2019 | Stereo-electroencephalography and thermocoagulation in drug-resistant epilepsy | European Journal of Neurology | 10.1111/ene.14019 | SEEG/RFTC therapeutic, efficacy, safety, or subtype-specific study; no extractable later resective/ablative surgery outcome for the target analysis. |
| Review/background | Negida | 2019 | The efficacy of stereo-electroencephalography-guided radiofrequency thermocoagulation in patients with drug-resistant epilepsy-a meta-analysis of 296 patients | Stereotactic and Functional Neurosurgery | 10.1159/000501568 | Review, survey, or background article; not an original extractable cohort for the planned synthesis. |
| Ancillary/biomarker or technical | Nacher | 2021 | Validity measures of direct cortical stimulation for seizure onset zone localization | Epilepsia | 10.1111/epi.17079 | Ancillary biomarker, network, neuropsychological, safety, or technical study; no eligible later-surgery outcome data. |
| Near-miss SEEG-RFTC outcome | Mirza | 2021 | Radiofrequency Thermocoagulation in Refractory Focal Epilepsy: The Montreal Neurological Institute Experience | Canadian Journal of Neurological Sciences | 10.1017/cjn.2020.263 | SEEG/RFTC therapeutic, efficacy, safety, or subtype-specific study; no extractable later resective/ablative surgery outcome for the target analysis. |
| SEEG/surgery without RFTC | Huang | 2024 | Observation on the surgical efficacy of bilateral temporal lobe epilepsy confirmed by stereoelectroen-cephalography | Chinese Journal of Neurosurgery | 10.3760/cma.j.cn112050-20231016-00124 | SEEG diagnostic or epilepsy surgery outcome study without SEEG-guided RFTC as the index intervention. |
| Near-miss SEEG-RFTC outcome | Liang | 2026 | SEEG-guided RF-TC for drug-resistant epilepsy: a study on the effectiveness of epileptic network ablation | Chinese Journal of Neurology | 10.3760/cma.j.cn113694-20250703-00389 | SEEG/RFTC therapeutic, efficacy, safety, or subtype-specific study; no extractable later resective/ablative surgery outcome for the target analysis. |
| Conference abstract | Chipaux | 2017 | Stereo electroencephalography-guided radio frequency thermocoagulation in drug-resistant focal epilepsy: A new pediatric technique | Epilepsia | 10.1111/epi.13944 | Conference abstract or short meeting report; insufficient extractable full-text data. |
| Conference abstract | Chipaux | 2017 | Stereo electroencephalography-guided radiofrequency thermocoagulation in drug-resistant focal epilepsy: A new pediatric technique | European Journal of Paediatric Neurology | 10.1016/j.ejpn.2017.04.689 | Conference abstract or short meeting report; insufficient extractable full-text data. |
| Near-miss SEEG-RFTC outcome | Fasano | 2017 | Intracranial EEG-guided radiofrequency thermocoagulation for refractory focal epilepsy | Stereotactic and Functional Neurosurgery | 10.1159/000478281 | SEEG/RFTC therapeutic, efficacy, safety, or subtype-specific study; no extractable later resective/ablative surgery outcome for the target analysis. |
| Review/background | Ryvlin | 2021 | Insular seizures and epilepsies: Ictal semiology and minimal invasive surgery | Current Opinion in Neurology | 10.1097/WCO.0000000000000907 | Review, survey, or background article; not an original extractable cohort for the planned synthesis. |
| SEEG/surgery without RFTC | Zhang | 2024 | Outcomes of stereoelectroencephalography-guided surgery of drug-resistant epileptic spasms and the prognostic factors | Chinese Journal of Neurosurgery | 10.3760/cma.j.cn112050-20240511-00159 | SEEG diagnostic or epilepsy surgery outcome study without SEEG-guided RFTC as the index intervention. |
| Review/background | Mazhit | 2026 | Epileptogenesis and Epilepsy Treatment: Advances in Mechanistic Understanding, Therapeutic Approaches, and Future Perspectives | International Journal of Molecular Sciences | 10.3390/ijms27031175 | Review, survey, or background article; not an original extractable cohort for the planned synthesis. |
| Conference abstract | Hagiwara | 2021 | Stereo-EEG guided thermocoagulation | Clinical Neurology | 10.5692/clinicalneurol.61_supplement_S1 | Conference abstract or short meeting report; insufficient extractable full-text data. |
| Near-miss SEEG-RFTC outcome | Dai | 2023 | Optimized SEEG-guided three-dimensional radiofrequency thermocoagulation for insular epilepsy | Acta Neurochirurgica | 10.1007/s00701-022-05401-9 | SEEG/RFTC therapeutic, efficacy, safety, or subtype-specific study; no extractable later resective/ablative surgery outcome for the target analysis. |
| SEEG/surgery without RFTC | Chiarello | 2023 | Stereo-EEG tailored resection in a child with presumed perinatal post-stroke epilepsy: The complex organization of epileptogenic zone close to eloquent cortex | Epilepsy and Behavior Reports | 10.1016/j.ebr.2023.100616 | SEEG diagnostic or epilepsy surgery outcome study without SEEG-guided RFTC as the index intervention. |
| SEEG/surgery without RFTC | Liu | 2024 | Surgical treatment strategies of periventricular nodular heterotopia for children with epilepsy combined with or without other pathologies | Chinese Journal of Neurosurgery | 10.3760/cma.j.cn112050-20231014-00121 | SEEG diagnostic or epilepsy surgery outcome study without SEEG-guided RFTC as the index intervention. |
| Ancillary/biomarker or technical | Guo | 2024 | Study on the correlation between the degree of damage and efficacy of radiofrequency thermocoagulation in the treatment of epilepsy | Chinese Journal of Neurosurgery | 10.3760/cma.j.cn112050-20231013-00118 | Ancillary biomarker, network, neuropsychological, safety, or technical study; no eligible later-surgery outcome data. |
| Near-miss SEEG-RFTC outcome | Xie | 2024 | Efficacy and safety of stereoelectroencephalography (SEEG) guided radiofrequency thermocoagulation (RF-TC) in the treatment of paediatric drug-resistant epilepsy | Folia Neuropathologica | 10.5114/fn.2024.136413 | SEEG/RFTC therapeutic, efficacy, safety, or subtype-specific study; no extractable later resective/ablative surgery outcome for the target analysis. |
| Ancillary/biomarker or technical | Williamson | 2026 | Radiofrequency Thermocoagulation Lesion Characteristics Using the Cross-Bonding Stereoelectroencephalography Electrode Technique in Normal Saline and Egg White Tissue Models | Stereotactic and Functional Neurosurgery | 10.1159/000548518 | Ancillary biomarker, network, neuropsychological, safety, or technical study; no eligible later-surgery outcome data. |
| Ancillary/biomarker or technical | Bourdillon | 2016 | SEEG-Guided Radiofrequency Thermocoagulation (SEEG RF-TC): From in vitro and in vivo Data to Technical Guidlines | Stereotactic and Functional Neurosurgery | 10.1159/000448961 | Ancillary biomarker, network, neuropsychological, safety, or technical study; no eligible later-surgery outcome data. |
| Review/background | Bottan | 2023 | \| Radiofrequency-thermocoagulation in pediatric epilepsy surgery: a review and pooled analysis | Epilepsia | 10.1111/epi.17787 | Review, survey, or background article; not an original extractable cohort for the planned synthesis. |
| Conference abstract | Dimova | 2010 | Radiofrequency thermocoagulation (RFTC) of ictal onset zone be meansof stereoelectroencephalographic (SEEG) electrodes | Epilepsia | 10.1111/j.1528-1167.2010.02658.x | Conference abstract or short meeting report; insufficient extractable full-text data. |
| Conference abstract | Jean | 2016 | SEEG Guided Radiofrequency- Thermocoagulation: A Potential Method for Pre-Resection Minimal Invasive Therapy | Stereotactic and Functional Neurosurgery | 10.1159/000448961 | Conference abstract or short meeting report; insufficient extractable full-text data. |
| SEEG/surgery without RFTC | Yu | 2019 | Surgical treatment in patients with periventricular nodular heterotopia-related epilepsy | Epilepsia | 10.1111/epi.16336 | SEEG diagnostic or epilepsy surgery outcome study without SEEG-guided RFTC as the index intervention. |
| Near-miss SEEG-RFTC outcome | Wang | 2020 | One-Stage High-Density Focal Stereo-Array SEEG-Guided Radiofrequency Thermocoagulation for the Treatment of Pediatric Giant Hypothalamic Hamartomas | Frontiers in Neurology | 10.3389/fneur.2020.00965 | SEEG/RFTC therapeutic, efficacy, safety, or subtype-specific study; no extractable later resective/ablative surgery outcome for the target analysis. |
| Near-miss SEEG-RFTC outcome | Tan | 2021 | Efficacy of conformal radiofrequency thermocoagulation guided by SEEG based on three dimensional images for the treatment of epileptic foci in functional areas | Chinese Journal of Neurosurgery | 10.3760/cma.j.cn112050-20210406-00166 | SEEG/RFTC therapeutic, efficacy, safety, or subtype-specific study; no extractable later resective/ablative surgery outcome for the target analysis. |
| Near-miss SEEG-RFTC outcome | Zhang | 2023 | Primary observation of clinical efficacy of radiofrequency thermocoagulation guided by stereotactic electroencephalography for the treatment of refractory epilepsy | Chinese Journal of Neurosurgery | 10.3760/cma.j.cn112050-20221124-00547 | SEEG/RFTC therapeutic, efficacy, safety, or subtype-specific study; no extractable later resective/ablative surgery outcome for the target analysis. |
| Case report/small case | Li | 2024 | RELN gene-related drug-resistant epilepsy with periventricular nodular heterotopia treated with radiofrequency thermocoagulation: a case report | Frontiers in Neurology | 10.3389/fneur.2024.1366776 | Case report or very small case-based report; insufficient cohort-level data for quantitative synthesis. |
| Near-miss SEEG-RFTC outcome | Yan | 2025 | Whether radiofrequency thermocoagulation guided by stereotactic electroencephalography can benefit drug-resistant epilepsy in the elderly | Acta Epileptologica | 10.1186/s42494-025-00207-5 | SEEG/RFTC therapeutic, efficacy, safety, or subtype-specific study; no extractable later resective/ablative surgery outcome for the target analysis. |
| Near-miss SEEG-RFTC outcome | Jin | 2025 | Efficacy and safety of stereoelectroencephalography-guided 3D radiofrequency thermocoagulation for the treatment of drug-resistant epilepsy | National Medical Journal of China | 10.3760/cma.j.cn112137-20240925-02179 | SEEG/RFTC therapeutic, efficacy, safety, or subtype-specific study; no extractable later resective/ablative surgery outcome for the target analysis. |
| Review/background | Catenoix | 2018 | The combination of stereo-EEG and radiofrequency ablation | Epilepsy Research | 10.1016/j.eplepsyres.2018.01.012 | Review/background publication; used for contextual interpretation and citation searching but not eligible as an original clinical study. |
| Review/background | Bourdillon | 2020 | Surgical techniques: Stereoelectroencephalography-guided radiofrequency-thermocoagulation (SEEG-guided RF-TC) | Seizure: European Journal of Epilepsy | 10.1016/j.seizure.2019.01.021 | Review/technical background publication; used for contextual interpretation and citation searching but not eligible as an original clinical study. |
| Review/background | Bottan | 2025 | Radiofrequency-thermocoagulation in pediatric epilepsy surgery: A systematic review and pooled analysis of cases | Seizure: European Journal of Epilepsy | 10.1016/j.seizure.2025.01.012 | Systematic review/pooled analysis; used for contextual interpretation and citation searching but not eligible as an original clinical study. |
| Review/background | Castellano | 2024 | Stereoelectroencephalography-Guided Radiofrequency Thermocoagulation: Diagnostic and Therapeutic Implications | Brain Sciences | 10.3390/brainsci14020110 | Review/background publication; used for contextual interpretation and citation searching but not eligible as an original clinical study. |
| Review/background | Cossu | 2017 | Stereo-EEG-guided radiofrequency thermocoagulations | Epilepsia | 10.1111/epi.13687 | Review/technical background publication; used for contextual interpretation and citation searching but not eligible as an original clinical study. |
| Review/background | Bourdillon | 2018 | Stereo-electroencephalography-guided radiofrequency thermocoagulation in patients with focal epilepsy: A systematic review and meta-analysis | Epilepsia | 10.1111/epi.14584 | Systematic review and meta-analysis; used for contextual interpretation and citation searching but not eligible as an original clinical study. |
| Review/background | Pichardo-Rojas | 2025 | Stereoelectroencephalography-guided radiofrequency thermocoagulation for drug-resistant epilepsy: A meta-analysis | Epilepsia | 10.1111/epi.18524 | Meta-analysis; used for contextual interpretation and citation searching but not eligible as an original clinical study. |
| Review/background | Li | 2023 | Magnetic resonance-guided laser interstitial thermal therapy vs. stereoelectroencephalography-guided radiofrequency thermocoagulation in epilepsy patients with focal cortical dysplasia: a systematic review and meta-analysis | Frontiers in Neurology | 10.3389/fneur.2023.1241763 | Systematic review and meta-analysis; used for contextual interpretation and citation searching but not eligible as an original clinical study. |
| Conference abstract | Tugbiyele | 2023 | Stereoelectroencephalography-guided radiofrequency thermocoagulation in paediatric patients with refractory epilepsy: a single-center prospective cohort | Brain and Spine | NR | Conference abstract only; insufficient full-report methodological and outcome information for inclusion as an original clinical study. |
| Ineligible intervention | Iwasaki | 2025 | Volume-Based Stereotactic Radiofrequency Thermocoagulation for Drug-Resistant Focal Epilepsy: Preliminary Multicenter Report From Japan | Operative Neurosurgery | 10.1227/ons.0000000000001869 | RFTC was performed as a separate volume-based stereotactic procedure rather than through implanted SEEG electrode contacts; therefore, the intervention did not meet the prespecified SEEG-guided RFTC definition. |
| Ineligible intervention | Wu | 2014 | Efficacy and safety of a new robot-assisted stereotactic system for radiofrequency thermocoagulation in patients with temporal lobe epilepsy | Experimental and Therapeutic Medicine | 10.3892/etm.2014.1620 | Deep-EEG-guided stereotactic RFTC was reported, but thermocoagulation through SEEG electrode contacts could not be established; therefore, the intervention did not meet the prespecified SEEG-RFTC definition. |
| Ineligible intervention | Zhao | 2017 | Stereotactic bilateral transfrontal minimal radiofrequency thermocoagulation of the amygdalohippocampal complex for bilateral medial temporal lobe epilepsy: a retrospective study of 12 patients | Epileptic Disorders | 10.1684/epd.2017.0914 | Depth-electrode-guided stereotactic RFTC using a separate lesioning approach did not meet the prespecified definition of thermocoagulation through SEEG electrode contacts. |

The table lists the 93 reports excluded after full-text assessment and the primary prespecified reason for each exclusion. Exclusion categories comprised absence of an extractable outcome meeting the prespecified therapeutic, prognostic, safety, or downstream-treatment eligibility criteria (n = 23); ancillary, technical, or biomarker outcomes only (n = 15); review, survey, or background publication (n = 22); SEEG or epilepsy surgery without RFTC as the index intervention (n = 8); case report or very small case series (n = 7); ineligible intervention or outcome (n = 10); conference abstract or short report (n = 6); overlapping cohort (n = 1); and non-peer-reviewed preprint (n = 1).

**SUPPLEMENTARY TABLE S3. Characteristics of studies contributing to the primary later-surgery meta-analysis.**

| **Study** | **Subsequent procedure** | **n** | **Favorable-outcome definition** | **Favorable/total** | **Postoperative follow-up** |
| --- | --- | --- | --- | --- | --- |
| Dimova 2017 | Resection | 9 | Engel I | 4/9 | Variable; not all patients documented at ≥12 months |
| Bourdillon 2017 | Conventional epilepsy surgery | 91 | Engel I–II | 67/91 | At least 24 months |
| Chipaux 2019 | Resection | 30 | Engel I–II | 24/30 | Mean 20.1 ± 9.7 months |
| Moles 2018 | Anterior temporal lobectomy | 19 | Engel Ia | 14/19 | 12 months after lobectomy |
| Contento 2021 | Resection | 21 | Favorable surgical outcome; predominantly Engel I | 16/21 | At least 12 months |
| Du 2024 | Delayed excision surgery | 24 | Engel I–II | 16/24 | At least 12 months; overall 12–58 months |

**Note:** Chandra et al. reported 18 favorable outcomes among 29 downstream interventions, comprising 27 resective/disconnective procedures and two vagus nerve stimulation procedures. Because favorable outcomes were not separately reported for the 27 qualifying procedures, this cohort was excluded from the strict primary meta-analysis.
