# Supplementary material for: Subsequent epilepsy surgery after SEEG-guided radiofrequency thermocoagulation: Outcomes and prognostic value of treatment response—a systematic review and meta-analysis
Source: Neurosurg Rev. 2026 Nov 6;49(1):638. doi: 10.1007/s10143-026-04561-x (PMC13740120; doi:10.1007/s10143-026-04561-x)
Supplement: Supplementary file 12 [file 10143_2026_4561_MOESM12_ESM.docx]

Pub Med : 1059

((“epilepsy”[MeSH Terms] OR epilep*[tiab] OR seizure*[tiab])
AND
(“stereoelectroencephalography”[MeSH Terms] OR stereoelectroencephalograph*[tiab] OR “stereo-electroencephalograph*”[tiab] OR “stereo electroencephalograph*”[tiab] OR SEEG[tiab] OR “stereo-EEG”[tiab] OR “stereo EEG”[tiab] OR “depth electrode*”[tiab] OR “intracranial EEG”[tiab] OR “intracerebral EEG”[tiab] OR “invasive EEG”[tiab])
AND
(radiofrequenc*[tiab] OR “radio-frequency”[tiab] OR thermocoagulat*[tiab] OR thermoablat*[tiab] OR “thermo-ablat*”[tiab] OR lesion*[tiab] OR ablat*[tiab] OR coagulat*[tiab] OR RFTC[tiab] OR RF-TC[tiab] OR “RF TC”[tiab]))

Embase : 1873

(‘epilepsy’/exp OR epilep*:ti,ab OR seizure*:ti,ab)
AND
(‘stereoelectroencephalography’/exp OR stereoelectroencephalograph*:ti,ab OR ‘stereo-electroencephalograph*’:ti,ab OR ‘stereo electroencephalograph*’:ti,ab OR seeg:ti,ab OR ‘stereo-eeg’:ti,ab OR ‘stereo eeg’:ti,ab OR ‘depth electrode*’:ti,ab OR ‘intracranial eeg’:ti,ab OR ‘intracerebral eeg’:ti,ab OR ‘invasive eeg’:ti,ab)
AND
(radiofrequenc*:ti,ab OR ‘radio-frequency’:ti,ab OR thermocoagulat*:ti,ab OR thermoablat*:ti,ab OR ‘thermo-ablat*’:ti,ab OR lesion*:ti,ab OR ablat*:ti,ab OR coagulat*:ti,ab OR rftc:ti,ab OR ‘rf-tc’:ti,ab OR ‘rf tc’:ti,ab)

Scopus : 1427

TITLE-ABS-KEY(epilep* OR seizure*)
AND
TITLE-ABS-KEY(stereoelectroencephalograph* OR “stereo-electroencephalograph*” OR “stereo electroencephalograph*” OR SEEG OR “stereo-EEG” OR “stereo EEG” OR “depth electrode*” OR “intracranial EEG” OR “intracerebral EEG” OR “invasive EEG”)
AND
TITLE-ABS-KEY(radiofrequenc* OR “radio-frequency” OR thermocoagulat* OR thermoablat* OR “thermo-ablat*” OR lesion* OR ablat* OR coagulat* OR RFTC OR “RF-TC” OR “RF TC”)

Web of science : 1225

TS=(epilep* OR seizure*)
AND
TS=(stereoelectroencephalograph* OR “stereo-electroencephalograph*” OR “stereo electroencephalograph*” OR SEEG OR “stereo-EEG” OR “stereo EEG” OR “depth electrode*” OR “intracranial EEG” OR “intracerebral EEG” OR “invasive EEG”)
AND
TS=(radiofrequenc* OR “radio-frequency” OR thermocoagulat* OR thermoablat* OR “thermo-ablat*” OR lesion* OR ablat* OR coagulat* OR RFTC OR “RF-TC” OR “RF TC”)
